# Green synthesis and characterization of iron oxide nanoparticles for the removal of heavy metals (Cd^2+^ and Ni^2+^) from aqueous solutions with Antimicrobial Investigation

**DOI:** 10.1038/s41598-023-31704-7

**Published:** 2023-05-04

**Authors:** Abdelrahman Mohamed, R. R. Atta, Amna A. Kotp, Fatma I. Abo El-Ela, Hany Abd El-Raheem, Ahmed Farghali, Dalal Hussien M. Alkhalifah, Wael N. Hozzein, Rehab Mahmoud

**Affiliations:** 1 Department of Chemistry, Faculty of Science, Beni-Suef University, Beni-Suef, Egypt; 2 Department of Chemistry, Faculty of Science, Damietta University, Damietta, Egypt; 3 St. Petersburg State University, 7/9 Universitetskaya Nab., St. Petersburg, 199034 Russia; 4 Materials Science and Nanotechnology Department, Faculty of Postgraduate Studies for Advanced Sciences, Beni-Suef University, Beni-Suef, Egypt; 5 Department of Pharmacology, Faculty of Veterinary Medicine, Beni-Suef University, Beni-Suef, 62511 Egypt; 6 Environmental Engineering Program, Zewail City of Science and Technology, October Gardens, 6th of October City, Giza, 12578 Egypt; 7 Department of Biology, College of Science, Princess Nourah Bint Abdulrahman University, B.O. Box 84428, Riyadh, 11671 Saudi Arabia; 8 Botany and Microbiology Department, Faculty of Science, Beni-Suef University, Beni-Suef, Egypt

**Keywords:** Chemistry, Materials science

## Abstract

Clove and green Coffee (g-Coffee) extracts were used to synthesize green iron oxide nanoparticles, which were then used to sorb Cd^2+^ and Ni^2+^ ions out of an aqueous solution. Investigations with x-ray diffraction, Fourier-transform infrared spectroscopy, transmission electron microscopy, X-ray photoelectron spectroscopy, nitrogen adsorption and desorption (BET), Zeta potential, and scanning electron microscopy were performed to know and understand more about the chemical structure and surface morphology of the produced iron oxide nanoparticles. The characterization revealed that the main component of iron nanoparticles was magnetite when the Clove extract was used as a reducing agent for Fe^3+^, but both magnetite and hematite were included when the g-Coffee extract was used. Sorption capacity for metal ions was studied as a function of sorbent dosage, metal ion concentration, and sorption period. The maximum Cd^2+^ adsorption capacity was 78 and 74 mg/g, while that of Ni^2+^ was 64.8 and 80 mg/g for iron nanoparticles prepared using Clove and g-Coffee, respectively. Different isotherm and kinetic adsorption models were used to fit experimental adsorption data. Adsorption of Cd^2+^ and Ni^2+^ on the iron oxide surface was found to be heterogeneous, and the mechanism of chemisorption is involved in the stage of determining the rate. The correlation coefficient R^2^ and error functions like RMSE, MES and MAE were used to evaluate the best fit models to the experimental adsorption data. The adsorption mechanism was explored using FTIR analysis. Antimicrobial study showed broad spectrum antibacterial activity of the tested nanomaterials against both Gram positive (*S. aureus*) (25923) and Gram negative (*E. coli*) (25913) bacteria with increased activity against Gram positive bacteria than Gram negative one and more activity for Green iron oxide nanoparticles prepared from Clove than g-Coffee one.

## Introduction

One of the most pressing issues facing the global community today is the pollution of the natural environment^1^. Heavy metal pollution is the primary ecological problem that endangers human health, animal and plant life^2,3^. Natural elements that have a high atomic weight and a density that is at least five times that of water are considered heavy metals^4^. Such metals are commonly characterized as elements necessary in trace levels but are harmful in large proportions^5^. As a result of rapid industrialization and urbanisation, heavy metals have accumulated in the environment, and their mobility and transport rates in the environment have dramatically increased since the 1940s. Cadmium (Cd) has been recognized as a human carcinogen by the Environmental Protection Agency in the United States (USEPA), and have a negative impact on health and bone demineralization, either directly through bone degradation or as a result of renal failure^6^. Among the most significant sources of cadmium are melting operations, metal refineries, mining, and the photographic industry. Nickel (Ni) is a known human carcinogen, causing kidney and lung problems, skin dermatitis, gastrointestinal distress, and pulmonary fibrosis.

Consequently, some countries have strict limits on how much heavy metal can be in wastewater. This has prompted many researchers to develop a variety of technical remediation procedures in order to bring environmental contamination levels within the legal limit. Some treatment methods used to purify water such chemical precipitation, electrodialysis, ion exchange, ultrafiltration, chemical oxidation, reverse osmosis, reduction, and adsorption.^6^. Adsorption is a growing technology for removing heavy metals from wastewater because it provides design flexibility, high-quality treated effluent, and adsorbent renewability. in order to extract heavy metal ions from wastewater derived from industrial processes, researchers used a variety of adsorbents, including zeolites^7–10^, activated carbon^11–14^, carbon nanotubes^15–19^, graphenes and fullerenes^20–25^ and nanomaterials^26,27^. Nanomaterials are increasingly being utilized in the process of removing heavy metal ions from wastewater because of their unique properties, such as low toxicity, stability, large pore size, as well as a large specific surface area with large adsorption capacity. The adsorption performance of graphene oxide (GO) for heavy metal removal was evaluated by Wang et al.^28^ in batch testing using a variety of parameters, including solution pH, time, temperature, the presence of coexisting ions, and the concentration of the heavy metals. For Zn^2+^ removal, the findings revealed that the kinetic model “pseudo-second order” and isotherm model “Langmuir” were good fits for the adsorption process well, and the greatest Zn^2+^ adsorption capacity was 246 mg/g, demonstrating that GO was an excellent adsorbent for Zn^2+^. In order to adsorb Cd^2+^ and Co^2+^ in water, Zhao and colleagues^29^ employed a modified Hummers process to synthesize a few-layered graphene oxide nanosheets. This study showed that pH had a significant impact on the adsorption effect, and the existence of humic acid in an aqueous solution might make the adsorption of Cd^2+^ and Co^2+^ less effective. Cd ^2+^ and Co^2+^ have a relatively large adsorption capacity of 106.3 and 68.2 mg/g onto GO, respectively.

In recent years, zerovalent metal nanoparticles have shown their effective use in the treatment and remediation of contaminated water. Nanoscale zerovalent iron (nZVI) has reportedly attracted a significant amount of interest as a potential novel adsorbent for the treatment of a variety of heavy metals, including mercury (II), chromium (VI), copper (II), nickel (II), and cadmium (II). The remarkable adsorption performance of nZVI is due to its huge specific surface area and high reduction capacity^30^. The synthesis of zero-valent iron can be accomplished by a variety of physicochemical techniques; however, the non-toxicity and reusability of biogenic^31^ and green synthesis using plants^32^ has attracted the attention of researchers. In addition, the nZVI can be capped and stabilized through the use of plant extracts. Li et al.^33^ reported that an uptake capacity of 250 mg/g was reached with an average removal efficiency of Cu^2+^ of more than 96% under working conditions of 10.20 g/L nZVI and 100 min of stirring time. In another study, Li et al. showed that copper could be removed from an aqueous solution with 99% efficiency in the presence of other metals^34^. According to a study published in 2018^35^, Mahboub Saffari reported that the highest amount of Cd^2+^ ions that may be proposed to be removed by nZVI supported biochar was around 99.72% at an initial Cd^2+^ ions concentration of 70.78 mg/L, an adsorbent dose of 0.56 g/L, a pH of 6.92, and a contact time of 40.42 min.

Synthesis strategies for nZVI are classified as top-down or bottom-up^36^. Among the top-down strategies are vacuum sputtering^37^, decomposition of iron compounds^38^, and ball milling^36^. Bottom-up strategies, on the other hand, facilitate the formation of nanoparticles by chemical synthesis, which is often accomplished through the reaction of sodium borohydride with Fe^2+^ or Fe^3+^ salts. The primary drawbacks of top-down approaches are the need for specialized equipment, the high operating expenses, and the poor nZVI production. On the other hand, sodium borohydride, which is costly and toxic, is often used in bottom-up strategies. During the synthesis of nZVI, NaBH_4_ produces oxidized boron compounds that are hard to extract from the nZVI product. Therefore, iron nanoparticle production strategies that are inexpensive, ecologically friendly, and scalable are in high demand. It is hard to deny the importance of Rajender S. Varma’s work in this area^39–44^. Instead of relying on synthetic chemicals, natural extracts are used, with no need for high temperature or pressure during the reaction. Polyphenols and flavonoids derived from beverages such as g-Coffee, tea, and wine, as well as proteins, and vitamins are examples of natural product extracts^40,45,46^. These components are harmless, biodegradable, and can act as reducing and capping agents, boosting nanoparticle production while simultaneously minimizing aggregation^46^. Previously, several natural reducing agents have been used for the green synthesis of nZVI^47–52^. A green synthesis approach, where green tea extract was used as reducing agent and stabilizer, was employed by Fang Zhu and his colleagues^53^ to create nZVI, which was then used in the treatment of Cr(VI)-contaminated groundwater. At a pH of 5, a temperature of 303 K, and an nZVI dose of 0.4 g/L, nZVI efficiently removed Cr (VI) from groundwater with a removal efficiency of 94.7%. In another work, Leaf extracts from oaks, cherry leaves, and mulberries were utilized to get “green” nZVI adsorbents to remove As (III) and Cr (VI)^54^. It was found that the developed green nZVI had nano-sized particles in the range of 10–30 nm. Through the use of transmission electron microscope (TEM) and scanning electron microscope (SEM), it was discovered that nanoparticles were found to be round, stable, and not very agglomerated.

Iron nanoparticles have been produced by researchers using plant extracts^55,56^ and used for adsorption of heavy metal from an aqueous solution. Plant extracts are used as reducers because of the polyphenol content, which is thought to convert iron ions to zero-valent iron. Plant extracts like Clove and green Coffee have been used to synthesize iron nanoparticles^66^. Where, Clove contains Eugenol, also known as 4-allyl-2-methoxyphenol, which is thought to be the most abundant component of Clove extract. It functions as a reducing agent and a stabilizer of iron nanoparticles (Fe NPs). G-Coffee has several different types of bioactive compounds, including saponins, polysaccharides, flavonoids, alkaloids, and phenols. These compounds included in g-Coffee seed extract serve a dual purpose as metal reduction agents and capping agents during the synthesis process for iron nanoparticles.

Therefore, it is of great importance to researchers to create effective and efficient oxide nanomaterial using a green method. The utilization of plant extracts to create nanoparticles by the reduction of metallic salts, however, is one of the greener approaches. It is a straightforward, low-cost, environmentally friendly, and human-safe therapeutic application^32^. For the adsorption removal of heavy metals, green produced iron oxide nanoparticles with plant extracts have recently been employed^31^. These characteristics inspire us to create an appropriate water adsorbent that is environmentally beneficial^57^.

In addition, iron oxide nanoparticles have shown antimicrobial properties, as well as effective use in various biological applications such as tissue engineering, targeted drug delivery, and antibacterial treatment. Iron oxides are biodegradable, biocompatible, and possibly not dangerous to humans^58^. Fe oxides NPs' antibacterial properties are mostly determined by particle size and synthesis ingredients. Effective antibacterial activity against the studied bacterial samples was detected for Fe NPs prepared using corn plant extract^59^. Arokiyaraj et al.^60^ reported that Fe oxide NPs prepared from Argemone mexicana L. leaf extract limits Proteus mirabilis and Escherichia coli growth.

Unfortunately, green synthesized Fe NPs usually obtain an amorphous phase^61–63^. Amorphous iron is by far the most unstable form of iron of all the different types of iron^64^. However, forming stable and crystalline Fe NPs is essential for careful characterization and potential for various applications. The process of calcination is one method that can be used to enhance the performance of iron oxide. It is possible to produce Fe_3_O_4_ nanoparticles through the pyrolysis of FeO (OH)^65^, and calcination at 400 °C results in the formation of the highly crystalline Fe_3_O_4_ phase^66^. However, it is important to characterize calcined Fe NPs prepared by plant extract in order to gain a better understanding of the calcination of Fe NPs and the variations of Fe species and organic functional groups. In addition, low-temperature calcination has been demonstrated to be an economically viable, productive, and ecologically benign approach. However, calcination at low temperatures can generally be used to improve the characteristics of green iron oxide nanoparticles.

Therefore, Fe NPs prepared in this work using the extract from natural products (Clove and g-Coffee) were subjected to calcination at 550 °C to get stable and crystalline Fe oxide NPs. The produced iron oxide nanoparticles before and after calcination were characterized using different characterization tools. Iron oxide nanoparticles were applied to remediate Cd^2^^+^ and Ni^2+^ contaminated groundwater. Different models of isotherm and kinetic adsorption of Cd^2+^ and Ni^2+^ on green iron oxide nanoparticles were applied to obtain and find the most suitable model to explain the mechanism of Cd^2+^ and Ni^2+^ adsorption process on the iron oxide nanoparticles. Also, the antimicrobial activity of Fe NPs was investigated.

## Materials and methods

### Chemicals

Ferric nitrate (Fe (NO_3_)_3_.9H_2_O, MW = 404 g/mol, Oxford laboratory reagent, India) was used as a precursor for preparing iron oxide nanoparticles. Green Coffee and Clove (Egyptian market, Egypt) have been used to make an extract as a green reducing agent for iron (III) nitrate salt. Sodium hydroxide and ethanol (Piochem for Laboratory Chemicals, Egypt) were utilized without additional purification steps before usage. In all experiments, Milli-Q water was used.

### Extract preparation

The Clove and g-Coffee extract were prepared using the previously described method^67^. Briefly, Clove and g-Coffee, before being dried and ground into a fine powder, were washed twice with milli-Q water to remove any dust particles. The Clove and g-Coffee powder was then heated for 10 min in 250 ml milli-Q water at 100 °C. Following the completion of the cooling process, the supernatant was filtered using Whatman filter sheets to remove any impurities. After that, the extract solution was placed in a refrigerator and kept at a temperature of 4 °C. Within the first 15 days, it was utilized as a reducing and stabilizing agent.

### Iron oxide nanoparticles preparation

As previously reported^67^ the synthesis of iron oxide nanoparticles (IONPs) using extract of Clove or green g-Coffee was conducted. Briefly, after constructing an aqueous solution of ferric nitrate (5 ml of 0.3 M Fe (NO_3_)_3_.9H_2_O), 15 ml of the previously prepared extract were added. The mixture's pH was adjusted to 6 using 0.10 M NaOH. The synthesis was carried out for 10 h with stirring at 300 rpm at room temperature (~ 25 °C). The Fe NPs were then collected by centrifuging the content for 20 min at 13,000 rpm. The sample was then cleaned with deionized water three times and ethanol to remove any unreacted biomolecules before drying for 24 h at 40 °C^68^. The clean and dried Fe NPs are stored for further characterization^69^. The corresponding iron oxide nanoparticles were obtained by annealing the obtained Fe NPs at 550 °C for 2 h.

### Characterization

PANalytical X-ray diffractometer (Empyrean) was used to investigate the crystalline phase of the prepared nanoparticles at 40 kV and 35 mA using Cu Kα radiation to produce X-rays with a wavelength of 0.154 nm. a rate of 0.02° min^-1^ was used to scan the prepared samples were from 0° to 80° (2θ). Fourier-transform infrared spectroscopy (FTIR) of the prepared nanoparticles was investigated using a Bruker, Vertex 70 spectrophotometer. The FTIR spectra were recorded in the range of 400–4000 cm^−1^. The nanoparticles’ surface morphology was investigated using a scanning electron microscope (SEM) and transmission electron microscope (TEM). necessary intervals for measurements.

Using the Barret-Joyner-Haleda (BJH) method and an automatic gas sorption system (Quanta chrome NOVA) at 77.3 K, the surface area and pore size distribution were determined, respectively.

To determine the elemental makeup of the adsorbent and adsorbate, X-ray photoelectron spectroscopy (XPS, Kratos-England) with an Al-K X ray monochromatic source (h = 1486.6 eV) was utilised.Point of zero charge (PZC) of the prepared materials was investigated by adding 0.05 g from the prepared LDH to 25 mL of NaCl solution (0.01 M) at various pH (3.0–10.0). Then, the result solution was shaked for 24 h to attain the final pH. The difference between the final pH and initial pH was drown versus the initial solution pH. PZC is estimated from the initial pH at which the pH = 0^70^.

### Adsorption methodology

Batch experiments were used to establish the adsorption isotherm equilibrium**.** All adsorption experiments of heavy metal ions (Cd^2+^ and Ni^2+^) were carried out using 0.05 g adsorbent per 50 mL of metal ions (20 mg/L) aqueous solution in a 250 mL conical flask, and the pH was adjusted at 6.0. The prepared solution was stirred at 225 rpm at 25 °C in the thermostatic water bath shaker for 24 h. After that, the resultant suspension at 6000 rpm for 5 min was centrifuged, and the supernatant was collected. Factors such as sorbent dosage (0.01–0.15 g), initial metal ions concentration (20–300 mg/L), and contact time (5–300 min) that affect the adsorption equilibrium and adsorption kinetics were studied. According to the following equations, the adsorbents’ equilibrium adsorption capacity (${q}_{e}$, mg/g) and removal percentage (R, %) have been calculated.

$$q_e=\frac{\left({C_i}-{C_e}\right)*V}{m}$$

$$R\left(\%\right)=\frac{{C}_{i}-{C}_{e}}{{c}_{i}}*100$$

where ${C}_{i}$, and ${C}_{e}$ (mg/L) are the initial and equilibrium concentration of metal ions, respectively. V (L) is the solution volume, and $m$ (g) is the adsorbent weight.

### Adsorption isotherm models

Understanding the mechanisms of adsorption provides valuable information about the adsorption process and therefore allows optimization of the design of adsorbents and adsorption systems. The adsorption mechanisms have been studied in various methods, such as molecular dynamics, adsorbent characterization before and after adsorption, density functional theory calculation, and modeling adsorption data^71–73^. Isotherm models (the surfactant adsorbed per gram of the adsorbent at constant temperature) are convenient and widely used to determine the correlation that will produce the most accurate curves. Various isotherm models have been applied in the present work, such as Langmuir, Freundlich, Temkin, Dubinin–Radushkevich, Langmuir–Freundlich, Sips, Redlich-Peterson, Toth, Kahn, Baudu, and Fritz-Schlunder.

Langmuir isotherm model assumes solute is adsorbed with a finite number of identical sites creating monolayer adsorption. The model of the nonlinear Langmuir isotherm can be presented in the following equation:

$${q}_{e}={q}_{\mathit{max}}\left(\frac{{k}_{L}{C}_{e}}{1+{k}_{L}{C}_{e}}\right)$$

where ${q}_{e}$, and ${q}_{max}$ (mg/g) are the amount of solute adsorbed on the adsorbent surface at equilibrium and the maximum adsorption capacity, respectively. ${k}_{L}$ (L/mg) is the Langmuir constant.

Langmuir, in 1918, established the concept of a dimensionless equilibrium parameter (R) in an effort to classify the shape of the isotherms into groups. This parameter is as follows:

$$R=\frac{1}{1+{k}_{L}{C}_{e}}$$

When R = 0, the isotherm is irreversible; when R = 1, the isotherm is liner; when 0 < R < 1, the isotherm is favorable; finally, when R > 1, the isotherm is unfavorable.

Freundlich isotherm model assumes that the solute is simultaneously adsorbed on heterogeneous surfaces with a large number of different sites. The Freundlich model can typically be applied to non-ideal adsorption. The model equation is:

$${q}_{e}={k}_{F} ({{C}_{e})}^{1/n}$$

where ${k}_{F}$ is the Freundlich constant, and $1/n$ is an empirical constant that gives an indication of the system’s adsorption capacity. If n is equal to one, the isotherm is linear, and the adsorption sites are uniform (as in the Langmuir model); if n is less than one, the presence of additional adsorbate in the absorbent increases the free energies of further adsorption; and if n is greater than one, the added adsorbates are bound with weak free energies.

Temkin isotherm model operates under the presumption that the adsorption heat of solute decreases in a linear manner as the adsorbent surface coverage increases. Also, this isotherm assumes that the identical distribution of binding energies describes the adsorption up to maximum binding energy. The following equation can be used to give a description of the Temkin isotherm.

$${q}_{e}=\frac{RT}{{A}_{T}}\mathit{ln}{A}_{T}+\frac{RT}{{A}_{T}}Ln {C}_{e}$$

where $R$ (8.314 J/K.mol) is the universal gas constant, $T$ (K) is the temperature, $b$ is a constant related to the adsorption heat, and ${k}_{T}$ (L/mol) is Temkin equilibrium binding constant.

Dubinin–Radushkevich (D–R) isotherm model is generally utilized for the purpose of expressing the adsorption mechanism with the distribution of Gaussian energy onto heterogeneous and homogeneous surfaces. This adsorption model is based on the pore-filling mechanism and is applied in the physical adsorption system (multilayer adsorption). The model equation is:

$${q}_{e}=\left({q}_{max}\right)\mathit{exp}\left(-{k}_{ad}{\varepsilon }^{2}\right)$$

$$\varepsilon =RT\mathit{ln}\left(1+\frac{1}{{C}_{e}}\right)$$

where ${k}_{DR}$ (mol^2^/kJ^2^) is Dubinin–Radushkevich isotherm constant, and $\varepsilon$ is the adsorption potential.

Langmuir–Freundlich isotherm model is used to describe the adsorption of solute and its distribution on heterogeneous surfaces. This model transforms into the Freundlich isotherm model when there is a low concentration of adsorbate, but when there is a large concentration of adsorbate, it transforms into the Langmuir isotherm. A general equation for this model can be written as the following:

$${q}_{e}=\frac{{q}_{max}{\left({k}_{LF}\,{C}_{e}\right)}^{n}}{1+{\left({k}_{LF }\,{C}_{e}\right)}^{n}}$$

where ${k}_{LF}$ is the equilibrium constant for a heterogeneous solid, and n is a heterogeneous parameter, and it lies between 0 and 1.

Sips isotherm model, which incorporates the Langmuir–Fernandlich model, is used to predict adsorption in heterogeneous systems. Using the Sips isotherm, the Freundlich isotherm limit associated with increased adsorbate concentration can be overcome. However, this model transforms into the Freundlich model at low adsorbate concentrations, and at high adsorbate concentrations, it is reduced to the Langmuir model (monolayer adsorption). The nonlinearized form of the Sips model is presented in the following equation:

$${q}_{e}=\frac{{\left({q}_{max}{k}_{s}\,{C}_{e}\right)}^{1/n}}{{\left(1+{k}_{s}\,{C}_{e}\right)}^{1/n}}$$

where ${k}_{s}$ (L/g) is Sips isotherm model constant, and $n$ (dimensionless parameter) describes the adsorbate-adsorbent system’s heterogeneity qualitatively.

Redlich-Peterson isotherm^74^ is a three-parameter adsorption isotherm commonly used as a compromise between the Freundlich and Langmuir systems and combines the beneficial aspects that are significant to both models. The Redlich–Peterson model can be presented in the following manner:

$$q_{e} = \frac{{k_{R } C_{e} }}{{1 + a_{R } C_{e}^{\beta } }}$$

where $k$ (L/g) and $a$ (L/mg) are the Redlich–Peterson isotherm constants, and β is an exponent that lies between 0 and 1.

Toth isotherm model is considered as a modified form of the Langmuir equation and is used to reduce the gap between the theoretical and experimental values. This model is used to describe the heterogeneous adsorption system at low and high concentrations of the adsorbate. The Toth isotherm model can be presented in this way:

$${q}_{e}=\frac{{k}_{e}\,{C}_{e}}{{\left(1+{\left({k}_{L}\,{C}_{e}\right)}^{n}\right)}^{1/n}}$$

where ${k}_{e}$ is isotherm constants, and ${k}_{L}$(mg/g), $n$ (mg/g) are Toth isotherm constants. Toth’s equation is converted to Langmuir’s equation at $n$ = 1. So, the parameter n tells us how heterogeneous the adsorption system is.

The Kahn isotherm model is an example of a general model that can describe the adsorption of biadsorbate from pure dilute liquids. For both single and multicomponent adsorption systems, this isotherm model was developed. Kahn’s isotherm model can be written as the following

$${q}_{e}=\frac{{q}_{max}{ b}_{k }{C}_{e}}{{\left(1+{b}_{k }{C}_{e}\right)}^{{a}_{k}}}$$

where ${b}_{k}$, ${a}_{k}$ are Khan isotherm model constants.

Baudu isotherm model is a modified form of the Langmuir isotherm model, where Baudu observed that at various equilibrium concentrations, the Langmuir coefficients calculated by tangent measurement aren’t constant across an extensive range. Therefore, the Langmuir model was transformed into the Baudu model as follows:

$${q}_{e}=\frac{{q}_{max}{ b}_{0}{ {C}_{e}}^{1+X+Y}}{1+{b}_{0}{ {C}_{e}}^{1+X}}$$

where ${b}_{0}$, $X$, $Y$ are constants. Baudu isotherm model can only be used in regions where (1 + x + y) 1 and (1 +  × 1) are less than 1.

Fritz-Schlunder isotherm model contains various coefficients in its form. Therefore, it is used to fit a wide range of adsorption experiment results. The model can be expressed by the following equation:

$${q}_{e}=\frac{{q}_{{max}_{Fss}}{k}_{1}{{C}_{e}}^{{m}_{1}}}{1+{k}_{2}{{C}_{e}}^{{m}_{2}}}$$

where ${k}_{1}$, ${k}_{2}$, ${m}_{1}$, ${m}_{2}$ are Fritz-Schlunder constants.

### Adsorption kinetic models

Any investigation of adsorption should begin with an analysis of the adsorption kinetics, as this is the most important factor to take into account. The study of adsorption kinetics explores the enhancements that occur in the sorption properties (the sorption mechanism and the reaction rate) of a system over time. In this context, the degree to which a surface is covered can provide information about the process rate. There are several different models that can be used to describe the adsorption kinetics. Analysis of the kinetic data of the adsorption of heavy metal ions (Cd^2+^ and Ni^2+^) onto iron oxide nanoparticles was performed in the present study by applying four kinetic models to find the best model to figure out which adsorption kinetics model best describes the process of adsorption.

A) Pseudo-first order model
  $$\mathrm{ln}\left({q}_{e}-{q}_{t}\right)=\mathrm{ln }{ q}_{e }- {\mathrm{ k}}_{1 }t$$
B) Pseudo-second order model
  $$\frac{\mathrm{t}}{{\mathrm{q}}_{\mathrm{t}}}=\frac{1}{{\mathrm{k}}_{2}{\mathrm{q}}_{\mathrm{e}}^{2}}+\frac{\mathrm{t}}{{\mathrm{q}}_{\mathrm{e}}}$$
C) Intraparticle diffusion model
  $${q}_{t}\equiv {\mathrm{k}}_{\mathrm{ip }}\sqrt{\mathrm{t}}+{\mathrm{C}}_{\mathrm{ip}}$$
D) Avrami model
  $$\mathrm{ln }\{\mathrm{ln }\left[{\mathrm{q}}_{\mathrm{e}}/\left({\mathrm{q}}_{\mathrm{e}}-{\mathrm{q}}_{\mathrm{t}}\right)\right]\}=\mathrm{n\,ln\,k}+\mathrm{n\,ln\,t}$$

where ${q}_{e}$ and ${q}_{t}$ are the amounts of metal ions adsorbed (mg/g) at equilibrium and time t (min), respectively, ${\mathrm{k}}_{1}$ (min^-1^) and ${\mathrm{k}}_{2 }(\mathrm{g}/\mathrm{mg min})$ are the rate constant of pseudo-first order and pseudo-second order models, respectively, ${\mathrm{k}}_{\mathrm{ip}}$ is the intraparticle diffusion rate constant ((mg/g min^1/2^)), C (mg/g) is the intercept, ${\mathrm{k}}$ is the Avrami rate constant, and $\mathrm{n}$ is the Avrami exponent (dimensionless).

### Functional errors analysis

In order to assess the reliability of the used isotherm and kinetic models, statistical evaluation was performed. Functional errors between experimental ${\mathrm{q}}_{\mathrm{e exp}}$ and calculated ${\mathrm{q}}_{\mathrm{e cal}}$ adsorption capacity were computed using three different equations.

- Correlation coefficient of determination (R^2^) = $\frac{\sum_{\mathrm{ i}=1}^{\mathrm{n}}{\left({\mathrm{q}}_{\mathrm{e\,cal}}^{\mathrm{i}} - {\mathrm{q}}_{\mathrm{e\,exp}}^{\mathrm{i}}\right)}^{2}}{\sum_{\mathrm{ i}=1}^{\mathrm{n}}\left[{\left({\mathrm{q}}_{\mathrm{e\,cal}}^{\mathrm{i}} - {\mathrm{q}}_{\mathrm{e\,exp},\mathrm{mean}}^{\mathrm{i}}\right)}^{2}\right]}$  
- Root mean square error (RMSE) = $\sqrt{\frac{1}{\mathrm{n}}\sum_{\mathrm{i}=1}^{\mathrm{n}}{\left({\mathrm{q}}_{\mathrm{e\,cal}}^{\mathrm{i}}-{\mathrm{q}}_{\mathrm{e\,exp}}^{\mathrm{i}}\right)}^{2}}$  
- Mean square error (MSE) = $\frac{1}{\mathrm{n}}\sum_{\mathrm{ i}=1}^{\mathrm{n}}{\left({\mathrm{q}}_{\mathrm{e\,exp}}^{\mathrm{i}}-{\mathrm{q}}_{\mathrm{e\,cal}}^{\mathrm{i}}\right)}^{2}$  
- Mean absolute error (MAE) = $\frac{1}{\mathrm{n}}\sum_{\mathrm{ i}=1}^{\mathrm{n}} \left|{\mathrm{q}}_{\mathrm{e\,exp}}^{\mathrm{i}}-{\mathrm{q}}_{\mathrm{e\,cal}}^{\mathrm{i}}\right|$  

### Recycling and stability of the prepared adsorbents

The chemical stability of prepared materials was performed using 0.10 g of adsorbent and was added into 200-ml aqueous solution at different initial pH (2.5–10) and shack for 24 h^75^. Then, the dissolved Fe^3+^ ions in the solution were detected using an atomic absorption spectrophotometer (model ZEISS-AA55, Germany).

For practical investigation, the recyclability of prepared adsorbents are important. The uptake process can become highly cost-effective, if the material is reused and recycled. In the present investigation, iron oxide was conducted by consecutive adsorption cycles of adsorption and desorption using diluted HCL (0.01 M) and then washed with bi-distilled water. The washed waste was dried at 60 °C for 24 h until complete dryness. The dried materials are immersed in Cd^2+^ or Ni^2+^ solution (20 mg/L) at pH 7. The waste was collected after the adsorption experiment, then washed several times and dispersed for another 120 min in a fresh metal ions solution (pH 7, 20 mg/L). This process was repeated five times to investigate the reusability of Iron oxide.

### Wastewater treatment

0.1 g of the prepared adsorbents was used to remove Cd^2+^ metal ions from the industrial effluent in Beni Suleiman, New urban communities’ body, New Beni Suef city development system in Beni-suef governorate, Egypt.

### Antimicrobial measurements

Gram positive (*S. aureus*) and Gram negative (*E. coli*) bacteria were selected for this experiment as representative bacteria for each type of bacterial classification in order to determine the efficacy of iron oxide nanoparticles prepared using Clove or g-Coffee extract as antimicrobial agents. The Cairo Microbiology Research Center provided the ATCC 25,913 and 25,923 strains of *E. coli* and *S*. *aureus* that were purchased. Additionally, Tylvalosin for Gram positive bacteria and Draxxin for Gram negative bacteria, both of which were purchased in pure form from *Pharma Swede Pharmaceutical Company*, were used to compare the effectiveness of iron oxide nanoparticles.

To assess the antimicrobial efficacy of iron oxide nanoparticles against the listed pathogens, the following method was used:. on each Muller-Hinton agar plate, 100 µl of each pathogen were aseptically disseminated using a sterile bench hockey stick, and the plates were then left on the bench for 30 min to allow the pathogens to pre-diffuse into the medium. The sterile discs were then impregnated overnight in twofold successive dilutions of iron oxide nanoparticles (1000, 250, 125, and 62.5 g/ml) and stacked on each plate using a sterile cork borer set of 5 mm. It is crucial to keep in mind that the conventional antibiotics stated above were used only as a point of reference. All plates were then incubated for 48 h at 37 °C.

It should be noted that the minimum inhibitory concentration (MIC) of the tested iron oxide nanoparticles prepared using Cove or g-Coffee versus the aforesaid microbes was determined employing Micro dilution test. In accordance with this method, a stock solution of iron oxide nanoparticles was prepared, and serially diluted into multiple sterilized tubes containing10^8^ CFU/ml of the tested bacteria that inoculated with Mueller Hinton Broth medium, which was favored; due to its ability to support the growth of most pathogens and its lack of inhibitors towards common antibiotics. For 24 h, all tubes were incubated at 37 °C. a tube of negative control broth, which contains the tested microorganism, and a tube of positive control broth, which contains the tested pathogen were both included for verification. It's important to note that the MIC was established as the lowest concentration that matched the positive control and had no discernible turbidity after evaluating the turbidity of the investigated tubes. In order to assess the minimal bactericidal concentration (MBC), at least two different concentrations of iron oxide nanoparticles dilutions were plated on Muller Hinton Agar Plate and counted to estimate the viable CFU/ml. The MBC is the lowest concentration of iron oxide nanoparticles at which no viable bacterial colonies are seen during a 24-h incubation period at 37 °C, demonstrating a predetermined reduction in CFU/ml (such as 99.9%) in comparison to the MIC dilution (bactericidal activity).

## Results and discussion

### Characterization of the prepared materials

**Figure 1:**
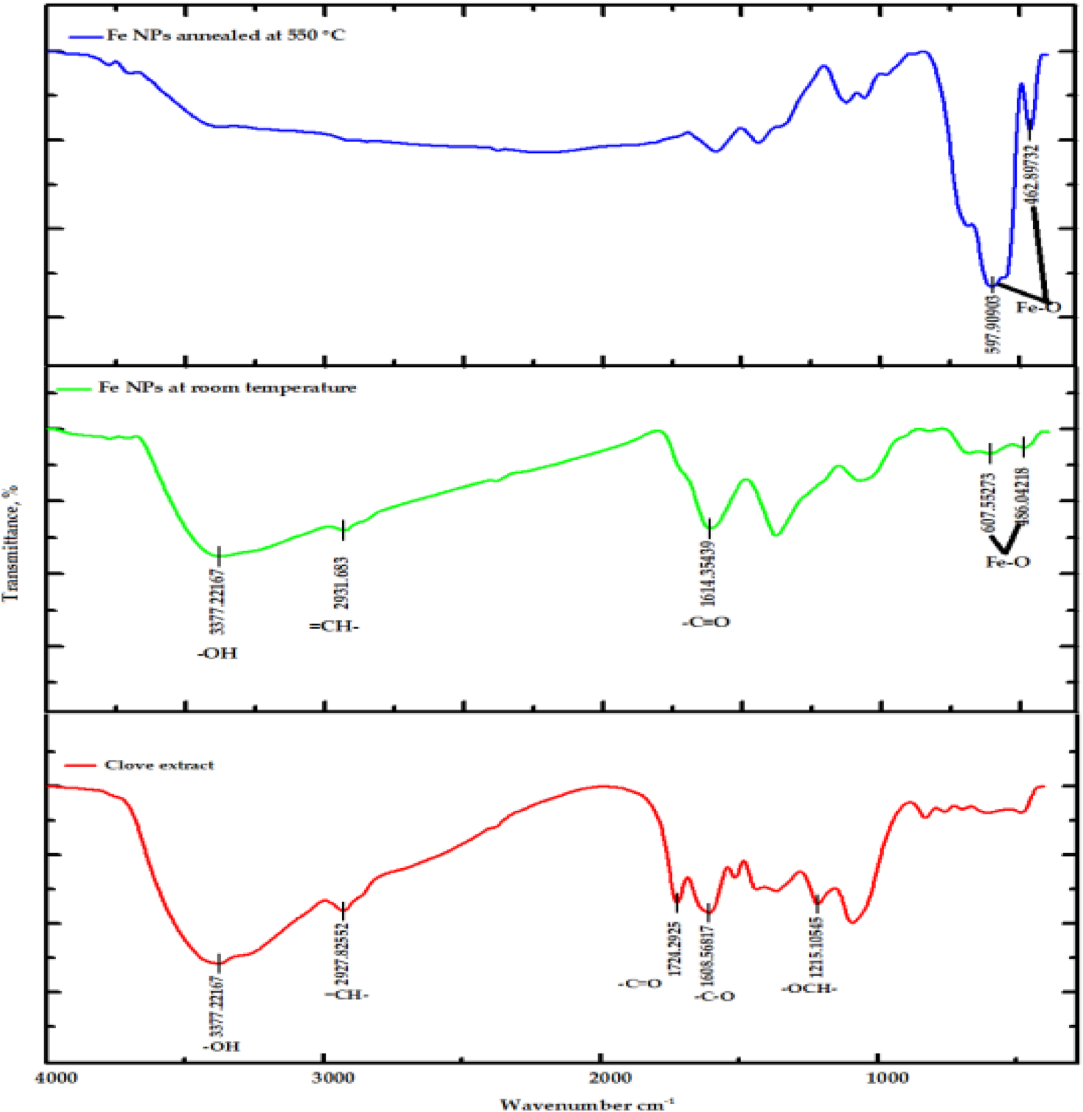
FTIR spectra of Clove extract and iron oxide nanoparticles prepared using Clove extract at room temperature and annealed at 550 °C.

**Figure 2:**
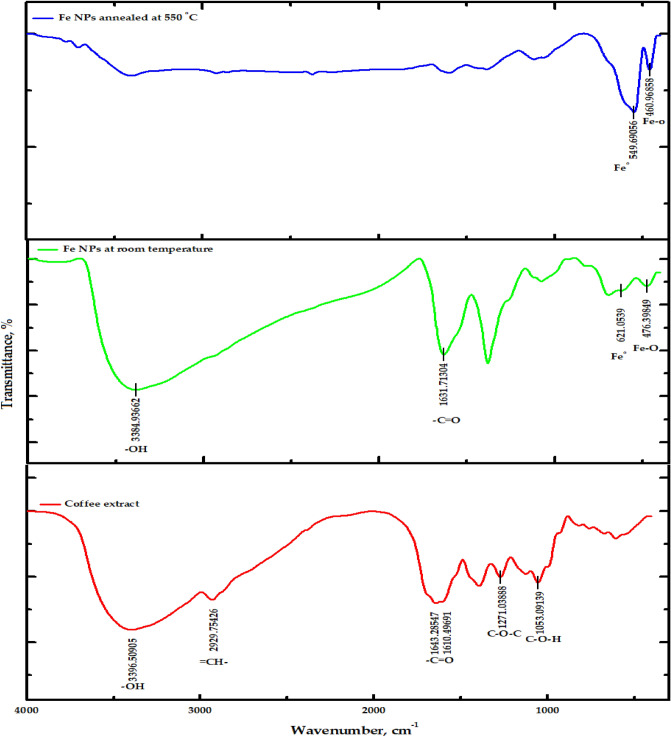
FTIR spectra of g-Coffee extract and iron oxide nanoparticles prepared using g-Coffee extract at room temperature and annealed at 550 °C.

The FTIR spectra of g-Coffee and Clove extract and iron oxide nanoparticles prepared at room temperature and annealed at 550 °C is presented in Figs. 1 and 2).

As obtained in Fig. 1, the spectrum of the Clove extract shows peaks at 3377, 2927, 1608, and 1215 cm^−1^, which correspond to –OH, =CH–, –C–O, and O–CH functional groups, respectively. These peaks indicate the presence of 4-Ally-2-methoxy-phenol (Eugenol), which acts as a reducing agent and stabilizer of Fe NPs in the Clove extract. In addition, the peak at 1724 cm^−1^, corresponding to the carbonyl group (C=O), indicates the presence of other secondary polyphenols (maybe acetyl eugenol)^76,77^. Also, the spectra of Fe NPs prepared using Clove extract at room temperature show the presence of Eugenol. Notably, the stretching band (O–CH) at 1215 cm^-1^ disappeared, and the carbonyl band was shifted from 1608 to1614 cm^−1^, indicating the coordination of Fe with the carboxylic group. When Fe NPs were prepared and annealed at 550 °C, peaks at 597 and 462 cm^−1^ were observed, indicating the presence of Fe–O groups associated with the hematite and magnetic core.

The g-Coffee extract’s FTIR spectrum obtained in Fig. 2 shows peaks at 3396, 2930, 1614, 1271, and 1053 cm^−1^ that are related to –OH, =CH–, C=O, C–O–C, and C–O–H functional groups, respectively. Observance of the bands around 1392, 1271, 1123, and 1053 cm^−1^ indicates the presence of polyphenolic acids like chlorogenic acids and caffeine that are responsible for the reduction of Fe (III) to Fe. The changes in the spectra of Fe NPs prepared using g-Coffee extract at room temperature, as well as the disappearance of the peak at 1271 cm^−1^, indicate the interaction of Fe nanoparticles with the components of g-Coffee extract. Also, when Fe NPs were prepared using g-Coffee extract and annealed at 550 °C, the peaks at 549 and 460 cm^−1^ that are related to Fe^0^ and Fe–O, respectively, indicate the existence of hematite and magnetite.

**Figure 3:**
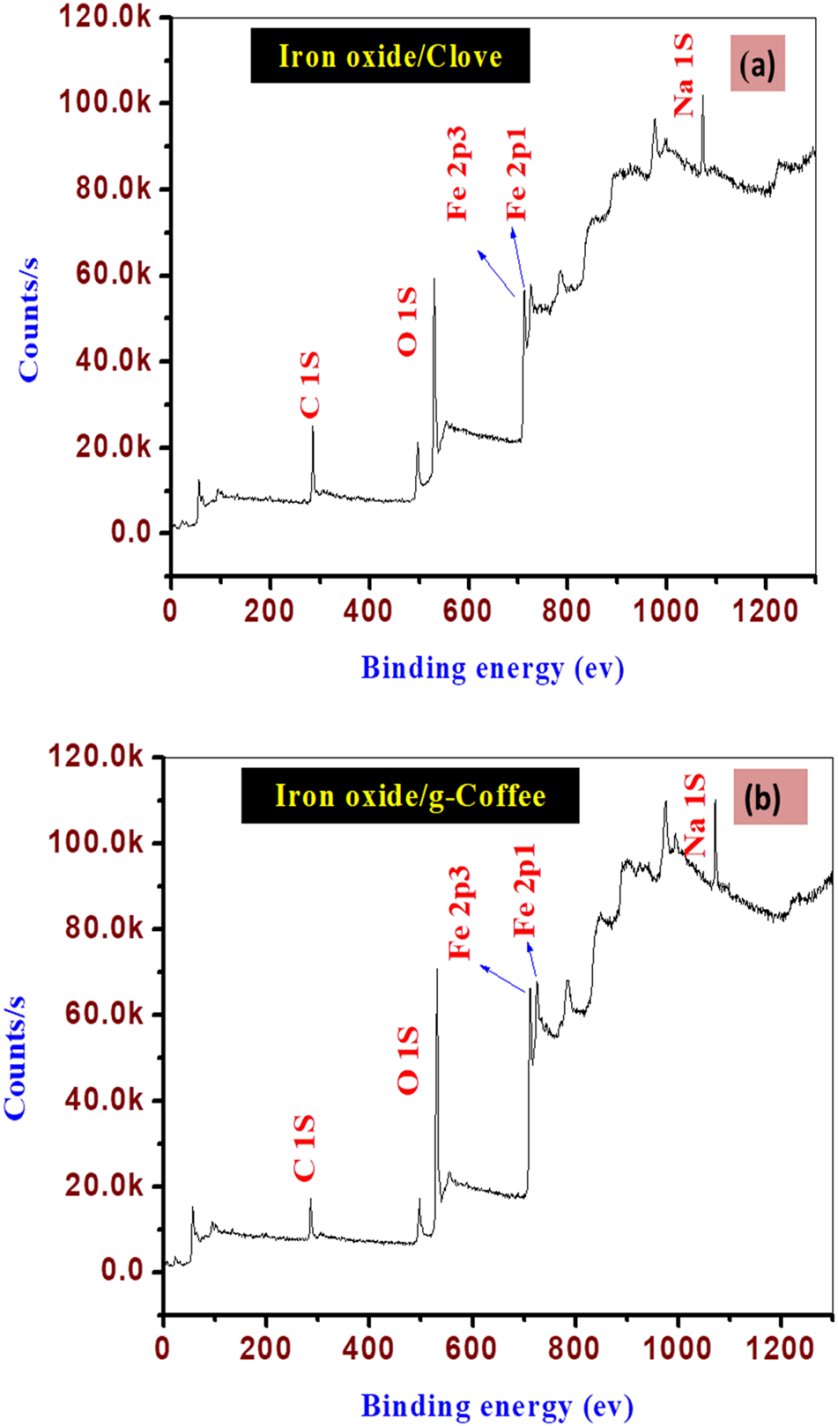
(**a**) XPS survey spectra of Iron oxide/Clove, (**b**) for iron oxide g-Coffee.

The chemical or elemental composition of as-prepared materials was assessed using XPS investigations (Fig. 3a,b). The presence of Fe, together with C and O, was plainly seen. Figure S(a-c) displays the deconvoluted O(1s) and C(1s) spectra of Fe_3_O_4_ nanoparticles, respectively. The O(1s) peaks at 530.42 and 534.87 eV result from O–H and C–O bonds that are formed by the components of adsorbed water and Clove extract, respectively. The C(1s) peaks at 285.69 and 288.10 eV are caused by C–C/C–H and C–O–C bonds, respectively, that are formed by extract molecules that have been chemically adsorbed to the surface of the particle. Additionally, the Fe–O bond of the Fe_3_O_4_ nanoparticles is responsible for the Fe(3p3/2), O(1 s), Fe(2p3/2), and Fe(2p1/2) peaks at about 55.69, 709.69, and 726.90 eV., respectively^78^ (Figure S(d–f)). A O(1s) core level spectra at 529.16 eVthat can be attributed to the presence of O–H groups attached to FeO. One peak, Fe–C at 284.6 eV, is one of the contributions to the C(1s) main envelope. The FeOH, COO, and COOH moieties are persistent organic groups on the surface following starch adsorption, and as a result, they must be connected to those that interact strongly with the oxy-hydroxide surface. XPS data support this hypothesis. Despite the difficulty in determining the exact type of chemical contact, the significant shift in the binding energy of Fe–OH points to a particular interaction, such as a coordinate link between the functional groups of the molecule and the FeO(OH)-like surface via oxygen atoms^79^.

**Figure 4:**
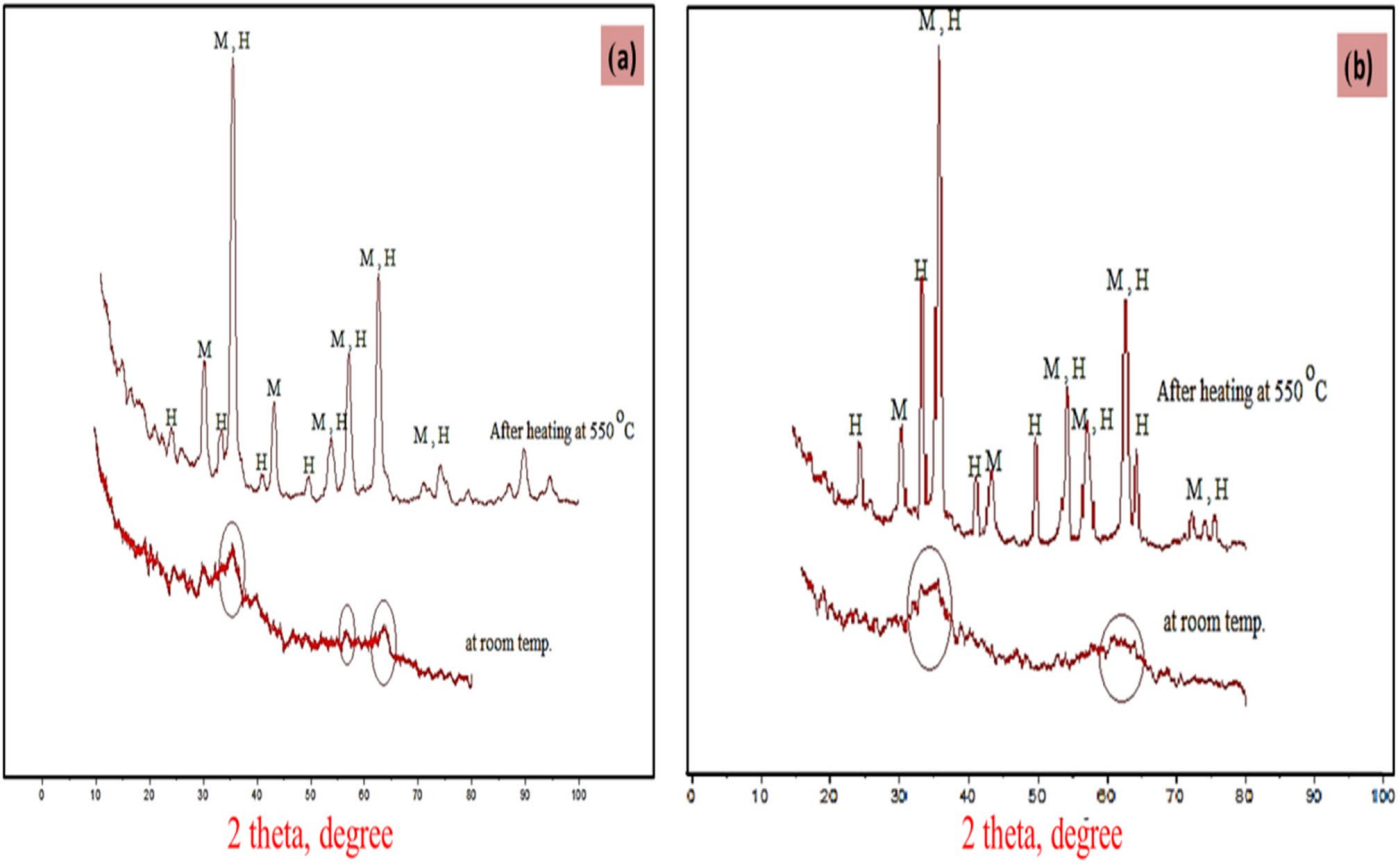
(**a**) XRD analyses of iron oxide nanoparticles prepared using Clove and (**b**) g-Coffee extract at room temperature and annealed at 550 °C.

The XRD analyses of Fe NPs prepared using Clove, and g-Coffee extract at room temperature and annealedat 550 °C. are presented in Fig. 4.

The iron nanoparticles prepared at room temperature exhibit weak diffraction peaks that are consistent with the standard diffraction of Fe–O powders (JCPDS File No. 74–1886). Other peaks have insufficient strength to be recognized, indicating that the prepared iron nanoparticles are amorphous. The XRD of iron oxide nanoparticles annealed at 550 °C confirm the formation of magnetite (M) and hematite (H). In the case of iron oxide nanoparticles prepared using Clove extract (at 550 °C), the intensity of the characteristic peaks attributed to hematite is weak, while that of magnetite is strong. This indicates that magnetite is the main component in the sample. On the other hand, X-ray diffraction analysis of iron oxide nanoparticles prepared using g-Coffee extract (at 550 °C) indicates the presence of magnetite and hematite.

**Figure 5:**
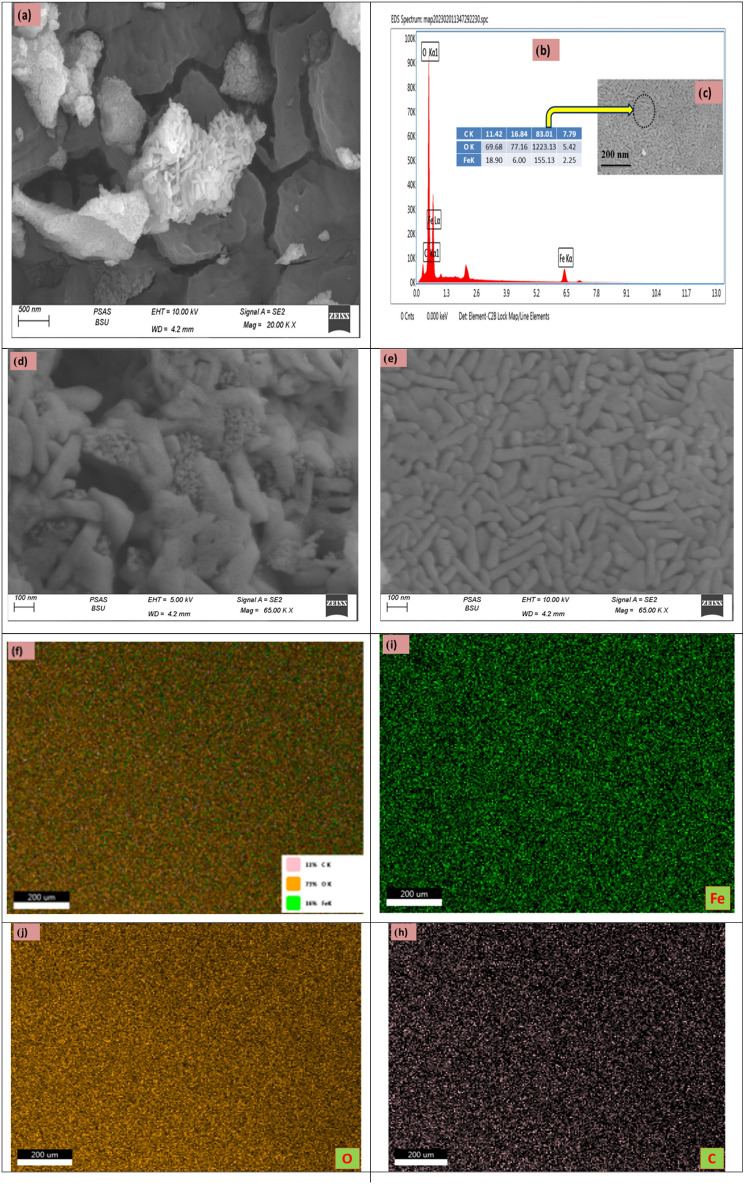
SEM images, EDS and the corresponding EDS elemental mapping C, O and Fe of the iron oxide prepared using Clove.

**Figure 6:**
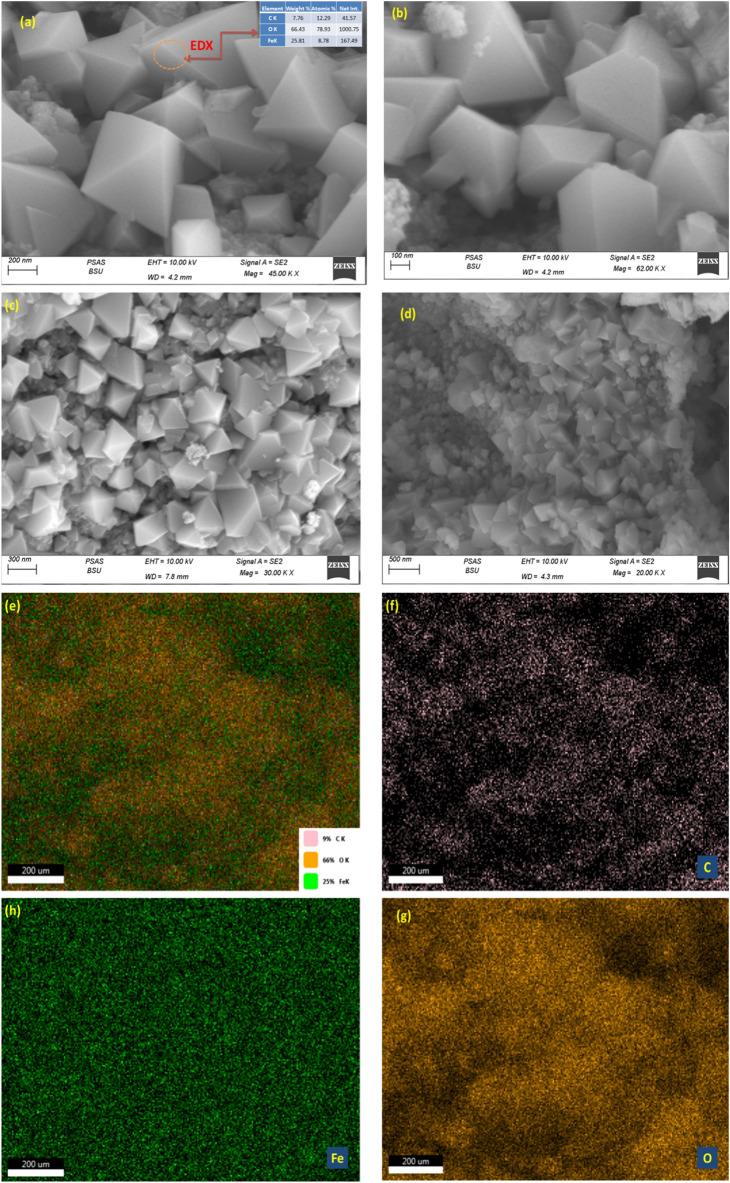
SEM images, EDS and the corresponding EDX elemental mapping C, O and Fe of the iron oxide prepared using g-Coffee.

The morphology of iron oxide nanoparticles prepared using Clove and g-Coffee extract was observed by SEM and presented in Figs. (5, 6).

It can be seen from SEM images (Fig. 5) that iron oxide nanoparticles prepared using Clove appear as rode shape morphology and also are agglomerated. The magnetic characteristics of iron particles should make it possible for iron oxide nanoparticles to exist in such close contact with each other, which matched with XPS and XRD. It has also been observed by Rehab et al.^67^ that iron oxide nanoparticles were agglomerated together. One possible explanation is that the presence of various polyphenols found in extracts have the potential to considerably alter iron oxide nanoparticle form and size^81,82^. In addition, the elemental distribution of C was steady with that of O (or Fe), suggesting that C was mainly appropriated by the iron oxide. EDX spectrum (Fig. 5b) confirmed the presence of target elements C, O and Fe species.

SEM images (Fig. 6a–d) showed that iron oxide nanoparticles prepared using g-Coffee appear as pyramidal shape morphology and also are clearly appeared large particles. In addition, the elemental distribution of C was steady with that of O (or Fe), suggesting that C was mainly appropriated by the iron oxide. EDX spectrum (Fig. 6a) confirmed the presence of target elements C, O and Fe species with notable differences in iron distribution in the prepared nanoparticles prepared using g-Coffee (Fig. 4f–h).

**Figure 7:**
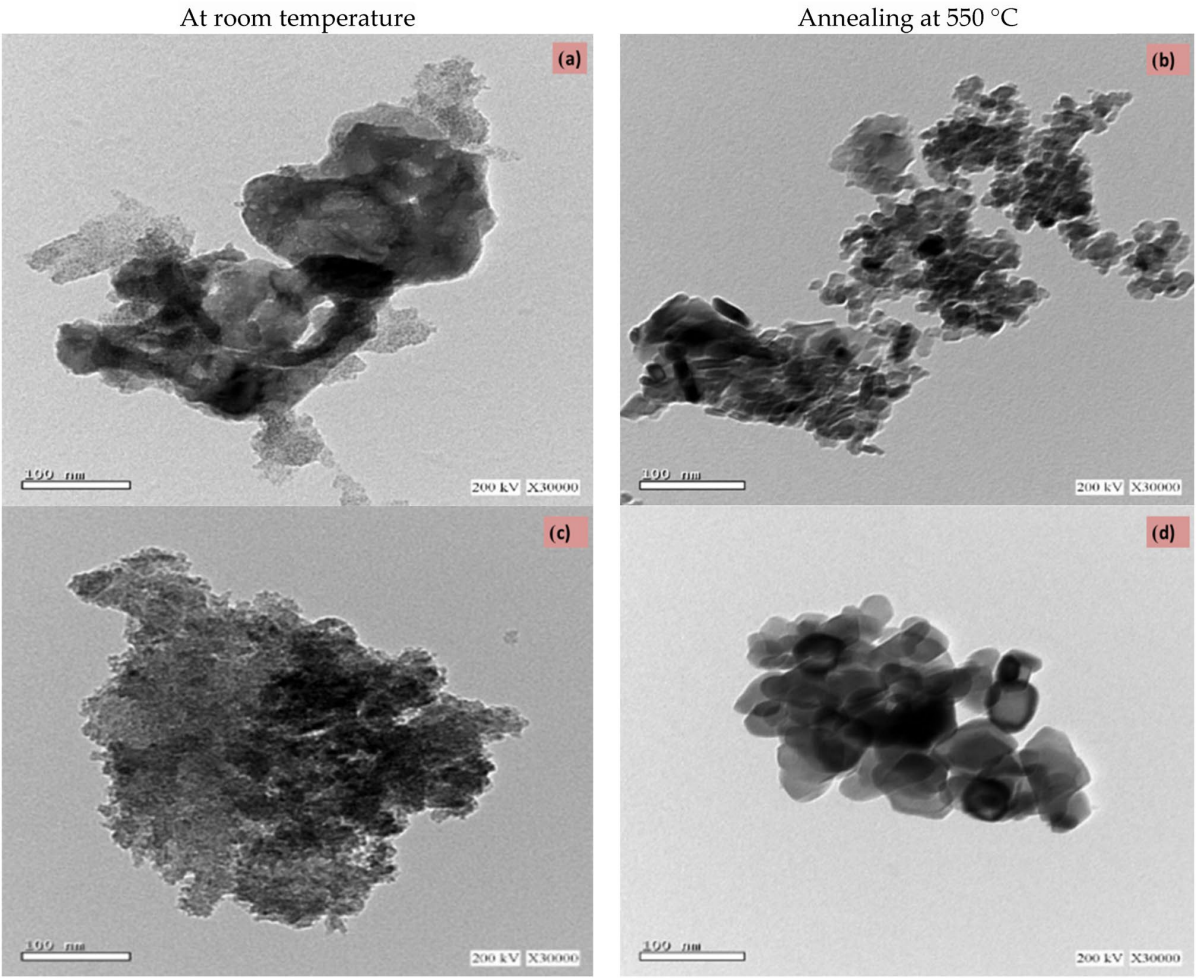
TEM images of iron nanoparticles prepared using Clove extract (**a**, **b**), and g-Coffee extract (**c**, **d**) at room temperature and annealed at 550 °C.

TEM analysis was performed to understand the morphology of iron and iron oxide nanoparticles prepared using Clove and g-Coffee extract in Fig. 7.

From the Fig. 7, Fe NPs prepared at room temperature using Clove or g-Coffee extract are agglomerated and have an amorphous structure. This may be due to Fe’s coordination with Eugenol in a Clove or with g-Caffeine in g-Coffee. on the other hand, Fe oxide have a crystalline structure, spherical partical and cubic shape like using Clove and g-Coffee extract, respectively. These results are compatible with X-ray and SEM analysis. It is also clear that the shape of the nanoparticles prepared with Clove differs from that prepared using g-Coffee. This suggests that the type of extract has an effect on the shape and the size of the nanoparticles. Potential to considerably alter iron oxide nanoparticle form and size^77,78^, the particle size distribution study from TEM images was performed using ImageJ, a freeware Java-based Image Processing program as shown in Figure S(2). Images show that the annealing of the particles at 550 °C led to narrow size distribution.

The textural characteristics are summarized in Table 1. The BET surface areas of iron oxide prepared using Clove and g-Coffee are 18.18 m^2^/g and 23.90 m^2^/g, respectively. These results show a dramatic decrease in the BET surface area of oxide prepared using Clove. This decrease in the surface area may be related to the dispersion of metal oxide and the aggregation of oxide which appear through the images of SEM images.

**Table 1:** Textural parameters of iron oxide prepared using Clove and g-Coffee.

Sample	S_BET_ (m^2^·g^−1^)	Total pore volume (cm^3^/g)	Average pore size (nm)
Iron oxide/Clove	18.18	0.0256	5.7905
Iron oxide/g-Coffee	23.90	3.7748	6.3171

### Adsorption conditions

#### Effect of pH

**Figure 8:**
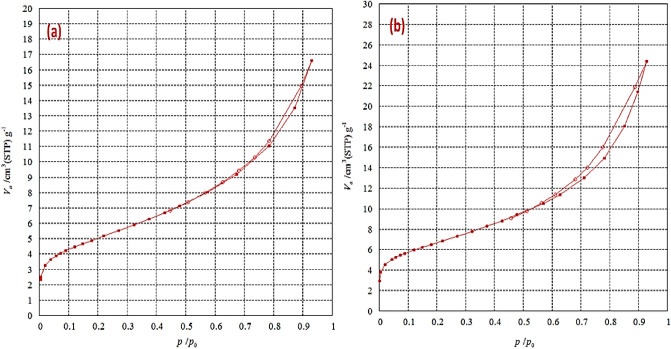
The nitrogen adsorption/desorption isotherms of iron oxide prepared using Clove (**a**) and g-Coffee (**b**).

The pH of the solution is one of the most important factors that affect the removal of heavy metals from aqueous solution by iron oxide nanoparticles prepared using Clove or g-Coffee. The prepared iron oxides adsorb heavy metals from an aqueous solution by exchange reaction and through surface complexation reactions that are dependent on the solution pH. In contrast, the anionic species becomes present in high percent within the pH range of 3–7, suggesting that the inner-sphere surface complexation is the main reason as a driving force for Ni^2+^ or Cd^2+^ uptake by iron oxide^85^. The effect of pH on the removal percentage of Cd^2+^ and Ni^2+^ from an aqueous solution by iron oxide nanoparticles prepared using Clove or g-Coffee was studied. The results are presented in Fig. 8.

The results show a limited removal of Ni^2+^ at pH 3 about 25.77 and 15.17% by iron oxide nanoparticles prepared using Clove and g-Coffee, respectively. The removal increases gradually with the increasing of pH and reaches its maximum value (99%) at pH 7 for iron oxide prepared using Clove and 66.50 for iron oxide prepared using g-Coffee. To interpret these results, the effect of pH on the four following parameters should be considered: (i) the speciation of Ni^2+^ in solution as a function of pH: the metal speciation by hydrolysis reaction affects the adsorption process. at pH < 7 Ni^2+^ is the predominant nickel species but at pH > 7 different nickel hydroxide species begin to appear^86^.

**Figure 9:**
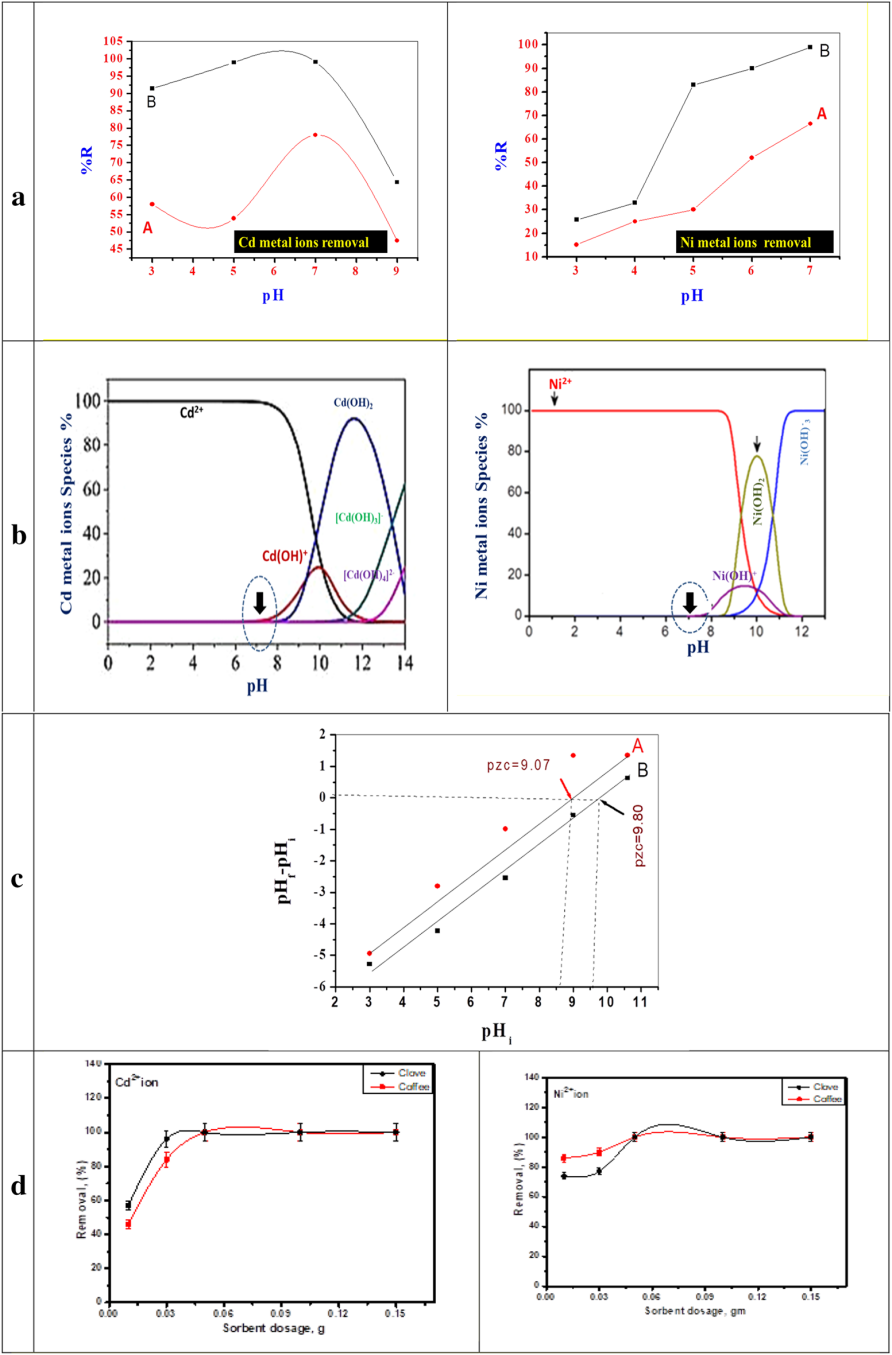
(**a**) The effect of pH on the adsorption of Cd^2+^ and Ni^*2*+^ by using iron oxide prepared using g-Coffee (A) and Clove (B), (**b**) species distribution curve of the Cd^2+^, Ni^2+^ metal ions at different pH respecivelly, (**c**) determination of the pH zero point charge and (**d**) the effect of sorbent dosage on the adsorption of Cd^2+^ and Ni^2+^.

The low metal removal percent at lower pH was due to the competition of metal ions with protons for active sites, as the active sites of adsorption on nanoparticles are protonated, and the statement justifies very less adsorption of Cd^2+^ ions at highly acidic pH^87^. Secondly, the protonation leads to electrostatic repulsion between the proton and metal ion too^85^. Since phenolics groups of plant extract responsible for fabrication were acidic in nature, the chance of alteration in fabricated surface of nanoparticles omitted at low pH^88^. At pH 7, the Cd^2+^ ions adsorption increased to 99.00% and 78.00% for iron oxide nanoparticles prepared using Clove and g-Coffee, respectively. The results were also confirmed using the species distribution curve of each pollutants in the aqueous solution at different pH, which shows that Ni or Cd metal ions present at pH as dissolved ions (Fig. 9).

The pH zero point charge (pHzpc) of iron oxide nanoparticles prepared using Clove or g-Coffee was measured at room temperature by solution addition method at pH range from 3 to 11, as shown in Fig. 9. The results demonstrated that both prepared oxides were negatively charged at pH (3–7) with notable stable dispersion, while they were negatively charged in the basic medium (pH = 10.70). The pHzpc value was 9.07 and 9.80 in both iron oxide prepared using g-Coffee and iron oxide prepared using Clove, which results in forming a negatively-charged surface as a dominant-negative charge at pH range 3–7 suggesting that prepared materials are suitable for heavy metal removal^89^.

#### Effect of sorbent dosage

The effect of the different weights of iron oxide nanoparticles prepared using Clove and g-Coffee on the removal percentage of Cd ^2+^ and Ni ^2+^ metal ions from an aqueous solution was studied. The results presented in Figure S(3) show that the adsorbent dosage has an impact on the removal percentage of Cd ^2+^ and Ni ^2+^ metal ions from the aqueous solution. Increasing the dose of the sorbent leads to greater removal of metal ions due to the greater number of active sites available. It was found that the adsorption of Cd ^2+^ onto iron oxide nanoparticles prepared using Clove is more than the adsorption of Cd ^2+^ onto iron oxide nanoparticles prepared using g-Coffee. But, the adsorption of Ni^2+^ onto iron oxide nanoparticles prepared using Clove is less than that of Ni^2+^ onto iron oxide nanoparticles prepared using g-Coffee. Also, the adsorption efficiency of Ni^2+^ is more than the adsorption efficiency of Cd^2+^. This can be explained by the magnetic properties of the metal ions (Cd^2+^ is a nonmagnetic divalent ion, whereas Ni^2+^ is a magnetic divalent ion).

#### Adsorption isotherms

**Figure 10:**
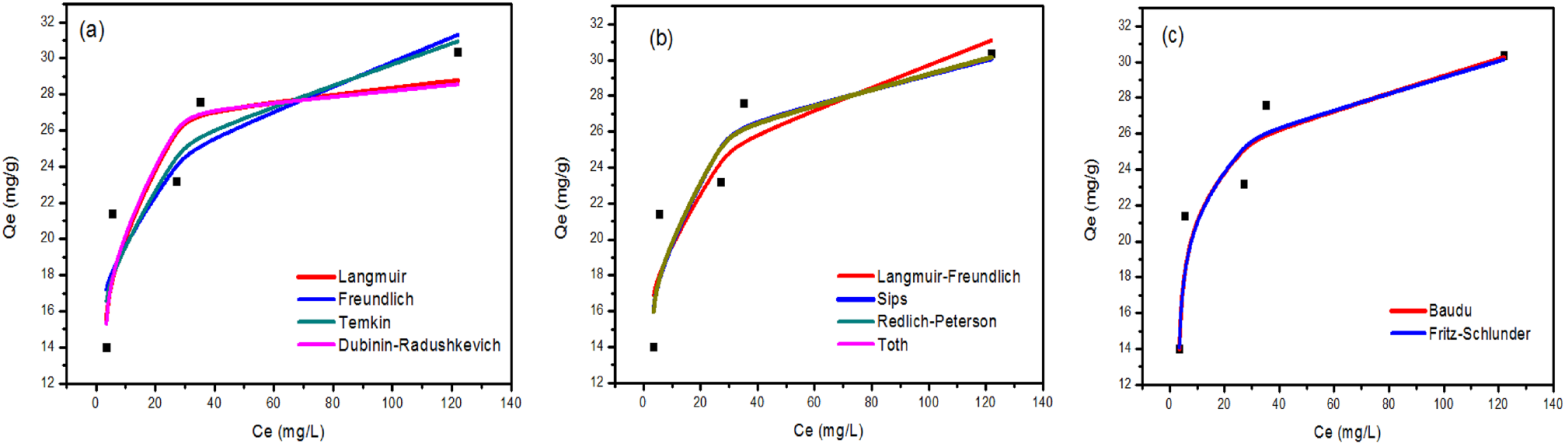
Isotherm plots for Cd^2+^ adsorption onto iron oxide nanoparticles prepared using Clove. (**a**) Two-parameter isotherm models, (**b**) three-parameter isotherm models, and (**c**) higher-parameter isotherm models.

**Figure 11:**
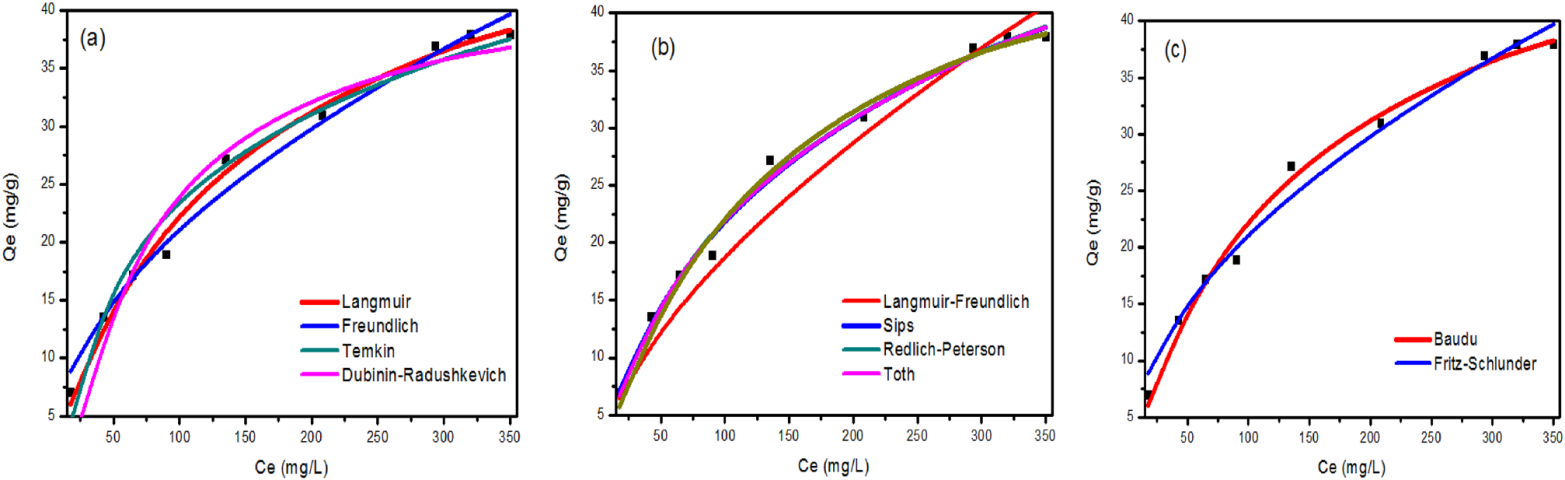
Isotherm plots for Ni^2+^ adsorption onto iron oxide nanoparticles prepared using Clove (**a**) Two-parameter isotherm models, (**b**) three-parameter isotherm models, and (**c**) higher-parameter isotherm models.

**Figure 12:**
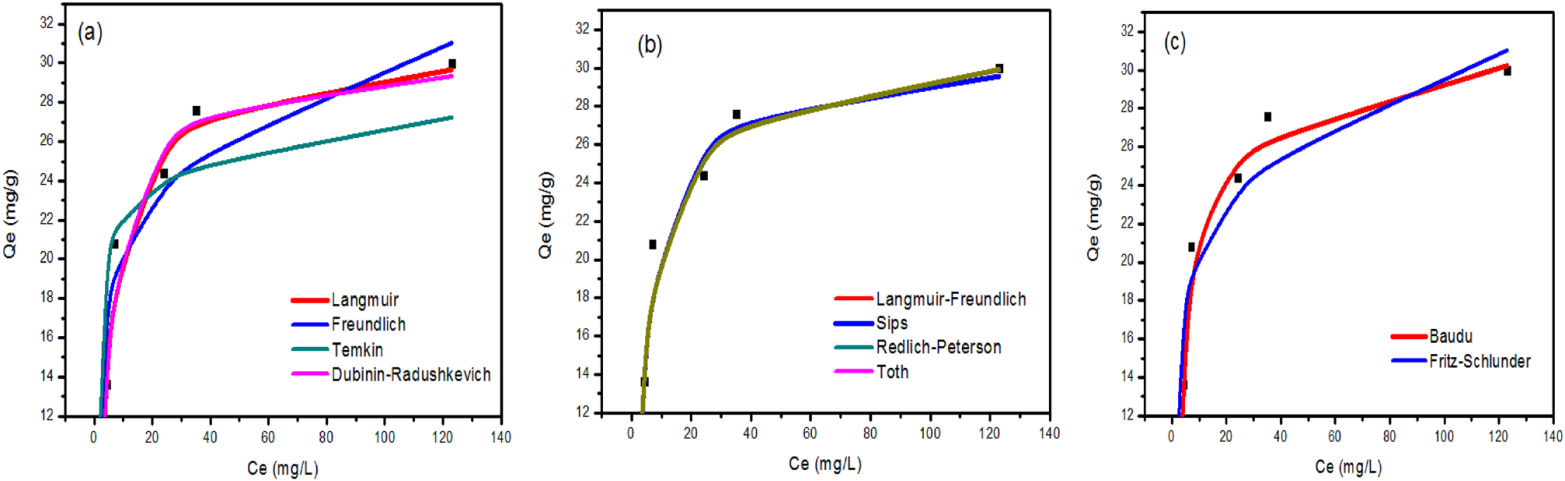
Isotherm plots for Cd^2+^ adsorption onto iron oxide nanoparticles prepared using g-Coffee. (**a**) Two-parameter isotherm models, (**b**) three-parameter isotherm models, and (**c**) higher-parameter isotherm models.

**Figure 13:**
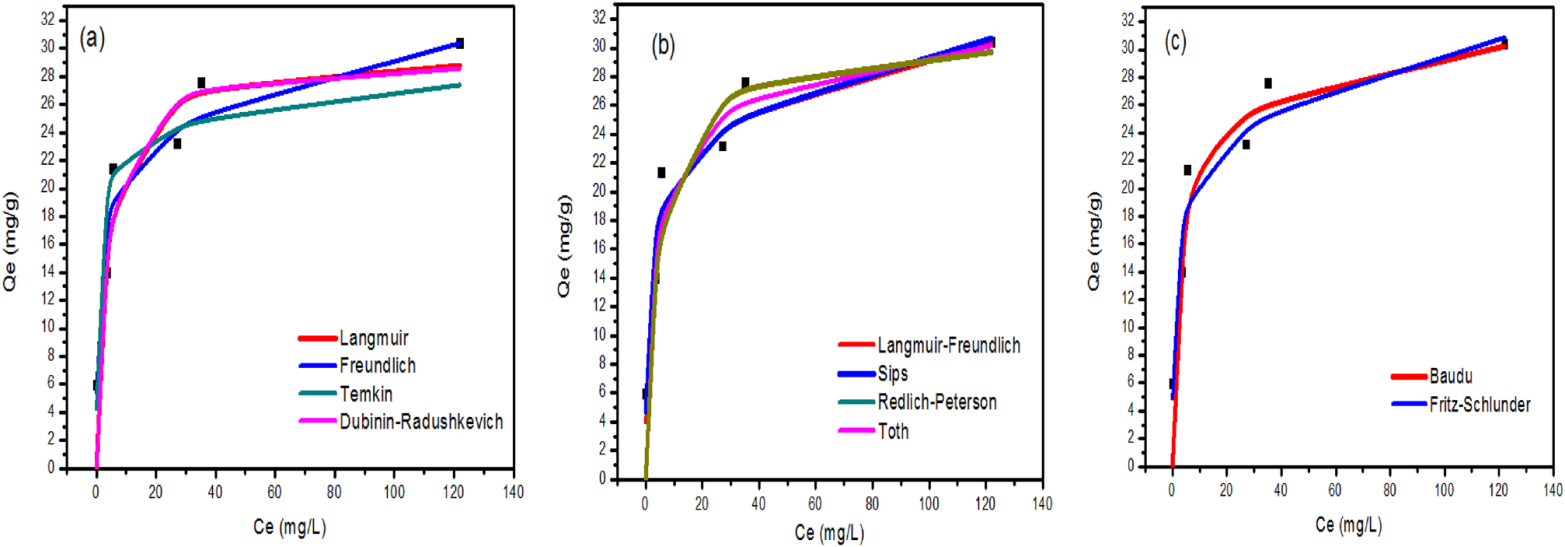
Isotherm plots for Ni^2+^ adsorption onto iron oxide nanoparticles prepared using g-Coffee. (**a**) Two-parameter isotherm models, (**b**) three-parameter isotherm models, and (**c**) higher-parameter isotherm models.

The effect of the initial concentration of metal ions on the adsorbed amount on the iron oxide nanoparticles was investigated and discussed (Figure S4). In this case, the total active adsorption sites on the sorbent surface are limited: at higher concentrations of metal ions, the available adsorption sites are already occupied, and in consequence, the adsorption of metal ions doesn’t occur, and the sorption capacity reaches equilibrium. However, this behavior has been reported in the most studied papers from the literature^83^. The isotherm data of q (mg/L) versus C (mg/L) were fitted to Langmuir, Freundlich, Temkin, Dubinin–Radushkevich, Langmuir–Freundlich, Sips, Redlich-Peterson, Toth, Kahn, Baudu, and Fritz-Schlunder models to describe the dynamics of sorption, including how adsorption quantity changes in response to initial metal ions concentrations, and how adsorbent and adsorbate are already in equilibrium with one another. Data for the adsorption of Cd^2+^ onto iron oxide nanoparticles prepared using Clove and g-Coffee extract are shown in Figs. 10 and 12), respectively. Similarly, Data for the adsorption of Ni^2+^ onto iron oxide nanoparticles prepared using Clove and g-Coffee extract are shown in Figs. 11 and 13) respectively.

Moreover, adsorption isotherm parameters are given in Table 2.

**Table 2:** Isotherm parameters of Cd^2+^ and Ni^2+^ adsorption onto iron oxide nanoparticles prepared using Clove and g-Coffee.

Model	Parameter	Cd^2+^		Ni^2+^	
		Clove	g-Coffee	Clove	g-Coffee
Langmuir isotherm	R^2^	0.85	0.89	0.99	0.86
	q_max_	29.53	30.77	53.50	29.53
	K_L_	0.317	0.220	0.007	0.318
Freundlich isotherm	R^2^	0.84	0.93	0.99	0.93
	1/n	0.17	0.16	0.50	0.15
	K_F_	13.917	14.075	2.099	14.980
Temkin isotherm	R^2^	0.86	0.83	0.98	0.85
	b_T_	614.69	1267.23	224.75	1261.22
	A_T_	16.855	8298.511	0.084	8503.456
Dubinin–Radushkevich isotherm	R^2^	0.86	0.89	0.94	0.86
	q_max_	29.17	30.19	43.43	29.17
	K_ad_	0.001	0.001	0.012	0.001
Langmuir–Freundlich isotherm	R^2^	0.85	0.89	0.96	0.93
	q_max_	67.85	30.53	1760.01	99.22
	K_LF_	0.004	0.221	0.000	0.000
	M_LF_	0.264	1.039	0.623	0.201
Sips isotherm	R^2^	0.87	0.89	0.99	0.93
	q_max_	34.50	30.53	68.17	290.84
	K_S_	0.43	0.21	0.01	0.05
	1/n	0.58	1.04	0.81	0.17
Redlich–Peterson isotherm	R^2^	0.88	0.89	0.99	0.87
	K_R_	14.52	7.14	0.49	14.70
	a_R_	0.68	0.25	0.03	0.70
	β	0.92	0.99	0.83	0.92
Toth isotherm	R^2^	0.88	0.89	0.99	0.87
	K_e_	13.41	7.04	0.41	13.55
	K_L_	0.68	0.25	0.03	0.70
	n	0.92	0.99	0.83	0.92
Kahn isotherm	R^2^	0.88	0.89	0.99	0.85
	q_max_	21.34	28.98	74.63	29.57
	b_K_	0.61	0.24	0.005	0.27
	a_K_	0.92	0.98	1.21	0.99
Baudu isotherm	R^2^	0.96	0.91	0.99	0.89
	q_max_	17.13	18.04	52.22	17.24
	b_0_	0.000	0.002	0.007	0.000
	x	0.12	0.11	0.003	0.12
	y	12.63	3.46	0.000	7.33
Fritz-Schlunder isotherm	R^2^	0.95	0.93	0.99	0.93
	q_maxFSS_	2.01	47.24	13.22	30.34
	K_1_	0.01	0.31	0.21	0.48
	K_2_	0.001	0.02	0.32	0.00
	m_1_	6.51	0.16	0.50	0.16
	m_2_	6.40	0.00	0.00	0.00

As illustrated in Figs. (10, 11, 12, 13) and Table 2, the adsorption of cadmium on iron oxide nanoparticles prepared using Clove can be described by Baudu isotherm model. The adsorption of cadmium on iron oxide nanoparticles prepared using g-Coffee can be described by Freundlich and Fritz-Schlunder isotherms. The adsorption of Nikel on iron oxide nanoparticles prepared using Clove can be described by Freundlich, Langmuir, Sips, Redlich–Peterson, Toth, Kahn, Baudu, and Fritz-Schlunder isotherms. The adsorption of Nikel on iron oxide nanoparticles prepared using g-Coffee can be described by Freundlich, Langmuir–Freundlich, Sips, and Fritz-Schlunder isotherms.

In addition, Four error estimation functions (R^2^, RMSE, MSE, and MAE) were taken into account in order to evaluate how well each isotherm model fitted the experimental data^84^ (Table 3).

**Table 3:** Comparison of statistical measures for isotherm model parameter optimization.

Model	Parameter	Cd (II)		Ni (II)	
		Clove	g-Coffee	Clove	g-Coffee
Langmuir isotherm	R^2^	0.85	0.89	0.99	0.86
	RMSE	2.13	2.74	1.03	2.13
	MSE	4.53	7.53	1.07	4.52
	MAE	1.91	1.97	0.90	1.84
Freundlich isotherm	R^2^	0.84	0.93	0.99	0.93
	RMSE	2.25	2.23	1.28	2.15
	MSE	5.06	4.98	1.64	4.62
	MAE	2.05	1.88	0.97	1.70
Temkin isotherm	R^2^	0.86	0.83	0.98	0.85
	RMSE	2.07	3.40	1.72	3.20
	MSE	4.30	11.57	2.95	10.23
	MAE	1.89	2.61	1.37	2.53
Dubinin–Radushkevich isotherm	R^2^	0.86	0.89	0.94	0.86
	RMSE	2.12	2.72	2.59	3.12
	MSE	4.48	7.40	6.73	9.73
	MAE	1.87	1.94	2.16	2.56
Langmuir–Freundlich isotherm	R^2^	0.85	0.89	0.96	0.93
	RMSE	2.15	2.74	2.29	2.22
	MSE	4.64	7.52	5.25	4.92
	MAE	1.97	1.97	1.89	1.92
Sips isotherm	R^2^	0.87	0.89	0.99	0.93
	RMSE	2.02	2.74	0.89	2.14
	MSE	4.10	7.52	0.79	4.60
	MAE	1.79	1.97	0.73	1.84
Redlich–Peterson isotherm	R^2^	0.88	0.89	0.99	0.87
	RMSE	1.95	2.74	0.92	3.02
	MSE	3.80	7.51	0.84	9.14
	MAE	1.72	1.94	0.76	2.43
Toth isotherm	R^2^	0.88	0.89	0.99	0.87
	RMSE	1.95	2.74	0.92	3.02
	MSE	3.80	7.51	0.84	9.14
	MAE	1.72	1.94	0.77	2.43
Kahn isotherm	R^2^	0.88	0.89	0.99	0.85
	RMSE	1.94	2.74	1.12	3.18
	MSE	3.76	7.50	1.26	10.10
	MAE	1.71	1.94	0.99	2.38
Baudu isotherm	R^2^	0.96	0.91	0.99	0.89
	RMSE	1.18	2.53	1.04	2.68
	MSE	1.39	6.40	1.07	7.19
	MAE	0.84	1.40	0.90	1.73
Fritz-Schlunder isotherm	R^2^	0.95	0.93	0.99	0.93
	RMSE	1.22	2.23	1.28	2.13
	MSE	1.49	4.98	1.64	4.52
	MAE	0.92	1.88	0.97	1.84

Table 3 shows that the highest R^2^ value and the lowest value for other error functions (means that the beast isotherms to describe the adsorption) were obtained for the Baudu isotherm, Freundlich and Fritz-Schlunder isotherms, Sips isotherm, and Fritz-Schlunder isotherm for the adsorption process of cadmium/Clove, cadmium/g-Coffee, Nickel/Clove, and Nickel/g-Coffee, respectively.

#### Sorption kinetic study

The effect of contact time between metal ions (Cd^2+^, Ni^2+^) and iron oxide nanoparticles prepared using Clove or g-Coffee is investigated and discussed (Fig. S5). An exceptionally high activity rate characterizes the first stage of the sorption process. In the first 60 min, more than 92% and 71% of Cd^2+^ and Ni^2+^ ions were removed by iron oxide nanoparticles, respectively. Following this initial stage, the rate of the sorption process gradually slows down until it reaches equilibrium. Finally, after almost one hour, there is no record of any more significant sorption. Therefore, this time is required to attain equilibrium. And this is because the sorbent surface at the beginning of the adsorption process has more sorption sites. On the other hand, the first stage of the adsorption of Cd^2+^ and Ni^2+^ ions onto iron oxide nanoparticles prepared using g-Coffee happened within 5 min, but the adsorption reached equilibrium after one hour. Also, the adsorption of Ni^2+^ ions onto iron oxide nanoparticles prepared using g-Coffee reached the first equilibrium within 5 min. After that, the adsorption started to increase again till it reached to the second equilibrium after 120 min, suggesting multilayer adsorption.

**Figure 14:**
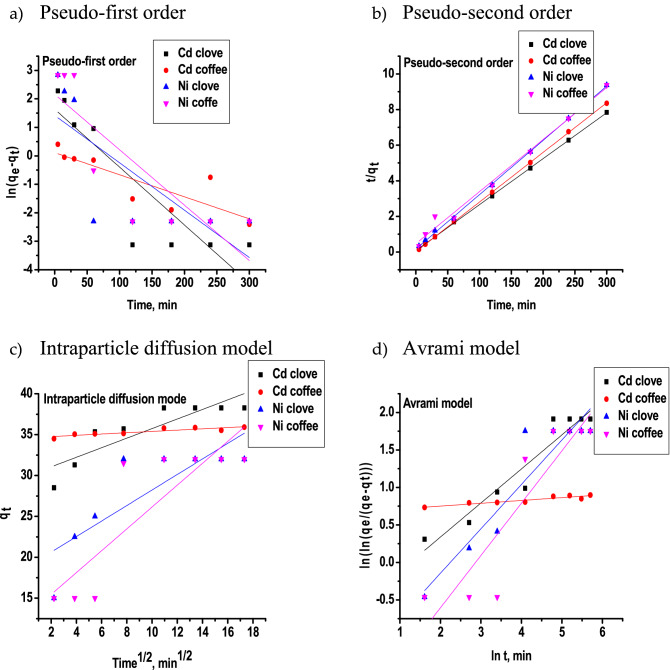
Fitting the adsorption kinetic models (Pseudo-first order, Pseudo-second order, Intraparticle diffusion model, and Avrami model) to the experimental adsorption data of heavy metal ions (Cd^2+^ and Ni^2+^) onto iron oxide nanoparticles prepared using Clove or g-Coffee.

For a better understanding of the adsorption kinetics and to find out the suitable mechanism that describes the adsorption of heavy metal ions (Cd^2+^ and Ni^2+^) onto iron oxide nanoparticles, the results gathered from the contact time experiments were fitted with four different adsorption kinetic models (Pseudo-first order, Pseudo-second order, Intraparticle diffusion model, and Avrami model). The plots are presented in Fig. 14. In addition, from these straight lines’ slopes and intercepts, model constants were determined and shown in Table 4.

**Table 4:** The kinetic adsorption parameters of heavy metal ions (Cd^2+^ and Ni^2+^) onto iron oxide nanoparticles prepared using Clove or g-Coffee obtained by liner equation of Pseudo-first order, Pseudo-second order, Intraparticle diffusion model, and Avrami model.

Model	Parameter	Cd^2+^		Ni^2+^	
		Clove	g-Coffee	Clove	g-Coffee
Pseudo-first order	R^2^	0.751	0.670	0.5128	0.672
	q_e_ cal	5.130	1.116	4.135	8.671
	K_1_	0.020	0.007	0.016	0.019
Pseudo-second order	R^2^	0.999	0.999	0.999	0.989
	q_e_ cal	38.759	35.855	32.776	34.246
	K_2_	0.0088	0.050	0.091	0.002
Intraparticle diffusion model	R^2^	0.731	0.772	0.630	0.682
	C_ip_	29.883	34.600	18.726	12.773
	K_ip_	0.585	0.079	0.949	1.337
Avrami model	R^2^	0.910	0.849	0.861	0.797
	n	0.454	0.037	0.592	0.697
	K_av_	3.509	83,741,333.54	0.106	0.056

Data shown in Fig. 14 and Table 4 reveals a low value of R^2^ for the Pseudo-first order, Intraparticle diffusion model, and Avrami model, obtaining that these models are not suitable to explain the adsorption mechanism of metal ions (Cd^2+^ and Ni^2+^) onto iron oxide nanoparticles. In comparison, the value of R^2^ (0.99) for Pseudo-second order indicates that this model best fits the experimental adsorption data. Therefore, it can be assumed that the mechanism of chemisorption is involved in the stage of determining the rate.

In addition, a variety of error functions may be found in the published research that can be used to determine whether or not the adsorption mathematical kinetic models are accurate representations of the experimental findings. In order to properly analyze kinetic data, it is not sufficient to rely solely on the regression coefficient (R^2^) because the outcomes of the experiments could have very high R^2^ values. Because of this, it is essential to diagnose the outcome of the regression before moving on to the residue analysis. In addition to the correlation coefficient (R^2^), additional validation of the applicability of the kinetic model to represent the adsorption process was provided by the RMSE, MSE, and MAE as show in Table 5.

**Table 5:** Statistics used to assess Kinetic model fit.

Model	Parameter	Cd (II)		Ni (II)	
		Clove	g-Coffee	Clove	g-Coffee
Pseudo-first order	R^2^	0.751	0.670	0.5128	0.672
	RMSE	13.58	21.31	9.41	14.19
	MSE	184.35	453.99	88.53	201.45
	MAE	9.67	18.21	7.36,	3.56
Pseudo-second order	R^2^	0.999	0.999	0.999	0.989
	RMSE	1.67	0.81	1.40	8.84
	MSE	2.81	0.65	1.97	78.12
	MAE	1.09	0.46	1.15	7.05
Intraparticle diffusion model	R^2^	0.731	0.772	0.63	0.682
	RMSE	1.67	0.20	3.37	4.27
	MSE	2.79	0.04	11.38	18.20
	MAE	1.50	0.18	2.62	3.56
Avrami model	R^2^	0.91	0.849	0.861	0.797
	RMSE	4.45	0.72	3.24	5.22
	MSE	19.83	0.52	10.48	27.21
	MAE	2.77	0.55	1.95	2.84

Table 5 shows that the Pseudo-second order model has the highest R^2^ value (0.999) and the lowest value for other error functions when comparing the experimental kinetics data and the calculated data for adsorption kinetics.

### Comparison of the adsorption capacity with other adsorbents

The adsorption capacity of Cd^2+^ and Ni^2+^ ions of different adsorbents are presented in Table 6 to obtain the possibility of using iron oxide prepared in this work as an adsorbent.

**Table 6:** Comparison of removing Cd, and Ni by different adsorbents.

Adsorbent	Adsorption capacity, mg/g		References
	Cd^2+^	Ni^2+^	
Iron oxide (Coffee)	74	80	This work
Iron oxide (Clove)	78	64.8	This work
Protonated rice bran		102.00	^90^
Turkish zeolite		119.7	^91^
Flyash		41.70	^92^
Blank alginate beads		24.4	^93^
Carbon aeroge (CAe, acid catalysis)	3.4	6.7	^94^
Montmorillonite		46.0	^95^
Purified MWCNTs		55.55	^96^
Aerogel carbon (acid catalysis)		12.8	^97^
Activated charcoal		99.0	^98^
Coir pith		15.95	^99^
Lignite		13.0	^100^
Walnut shell carbon		15.34	^101^
Aluminosilicates	42.7–57.9		^102^
Iron ore slime	34.75		^103^
Psidium guvajava L leaf powder	31.15		^104^
Rice husk	8.5		^105^
Rice husk treated with Na_2_CO_3_	16.1		^105^
Rice husk treated with NaOH	20,2		^105^
Activated carbon	19		^106^
Peanut hulls	5.96		^107^
Iron-Coated australian zeolite	3.7		^108^
acid-modified carbon-based adsorbents	1.22 to 2.02		^109^
Spent grain	17.3		^110^

As seen in Table 6, the iron oxide prepared in this work has a relatively higher maximum adsorption capacity for the Cd^2+^ and Ni^2+^ ions. Moreover, the sample pH (~ 6) and other operating parameters demonstrate that the present adsorbent is suitable for application in wastewater treatment.

### Mechanism of adsorption

**Figure 15:**
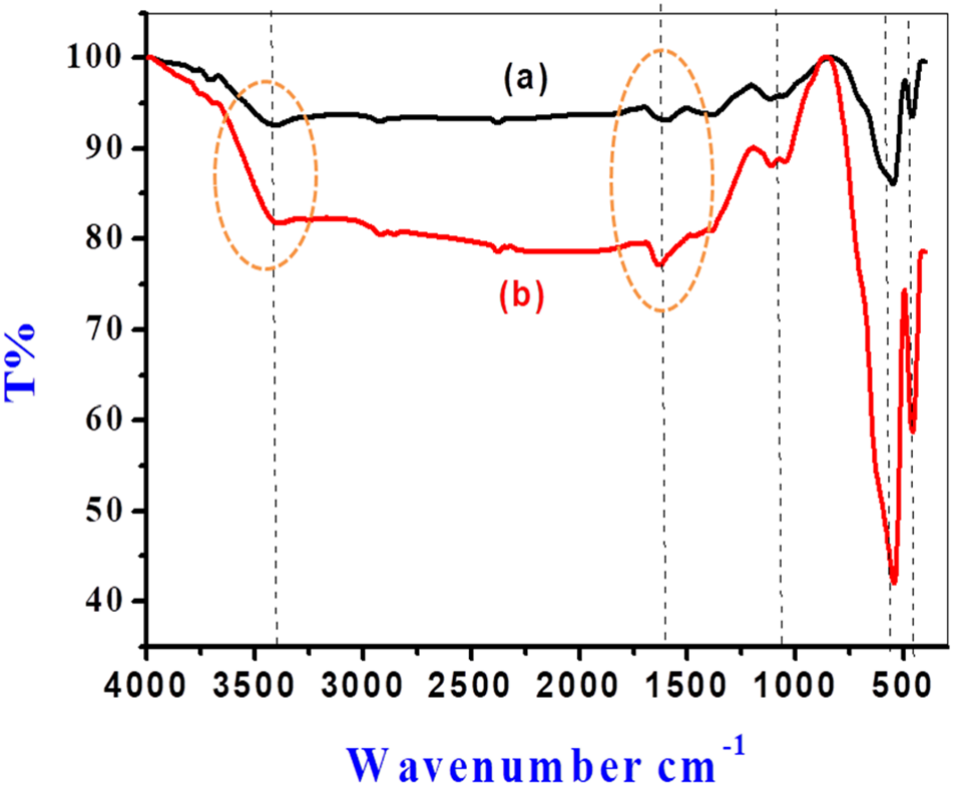
FTIR spectra of iron oxide prepared using g-Coffee (**a**) and iron oxide prepared using g-Coffee after Cd^2+^ sorption Cd^2+^ (**b**).

Figure 15 shows the FT-IR spectra of iron oxide prepared using g-Coffee (a) and iron oxide prepared using g-Coffee after Cd^2+^ sorption (b) as a representative example. The broad peak presented at around 3394.36 cm^−1^ is assigned to the O–H stretching vibration, indicating the presence of water molecules in both of the iron oxide prepared using g-Coffee and iron oxide prepared using g-Coffee after Cd^2+^ sorption. The strong absorption band at 1669 cm^−1^ is assigned to the bending vibration of hydroxyl groups^67^. The characteristic peak of the M–O bond at 537.50 cm^−1^ increased in intensity after Cd^2+^ sorption through chemical interaction between Cd^2+^ metal ions and oxygen atoms of iron oxide prepared using g-Coffee. The FT-IR spectrum of iron oxide prepared using g-Coffee and iron oxide prepared using g-Coffee after Cd^2+^ sorption has shown the change in the hydrogen bond intensity after adsorption (Fig. 15) via some change like the broadening of the –OH band at 3394.36 cm^−1^. This broadening might be attributed to the O–H bond stretching vibration of water molecules adsorbed on the oxide surface via hydrogen or chemical bonding with Cd^2+^ metal ions. The hydrogen bonding intensity was investigated from the ratio of the absorbance bands at 3394.36 cm^−1^ (for the –OH peak) and 1669 cm^−1^ (for the O–H peak) in oxide before and after adsorption respectively, showing a significant increase in the case of iron oxide prepared using g-Coffee after Cd^2+^ sorption (1.062) compared to that of iron oxide prepared using g-Coffee (0.99), and that confirmed the present of H- bonding interaction^89^.

### Stability and recycling study of the prepared adsorbents

**Figure 16:**
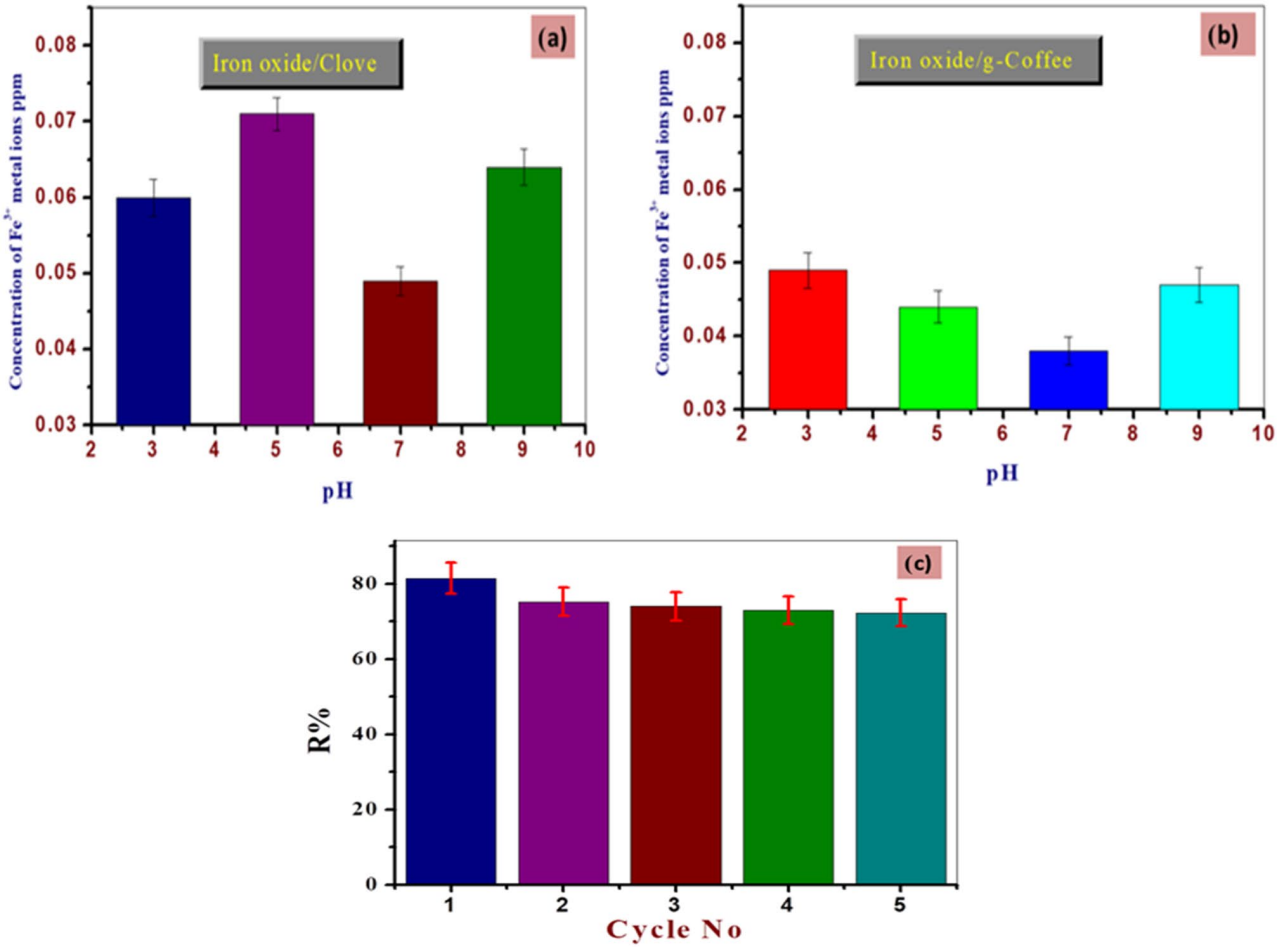
Stability of the used adsorbents at different pH and recycling study of the iron oxide prepared using Clove as a representative example.

Using an atomic adsorption spectrometer, the chemical stability of iron oxides was investigated by calculating the concentration percentages of iron in solution at various starting pHs (3–9). As shown in Fig. 16a,b, prepared iron oxides have good chemical stability at the studied pH range.

Also, it is important to control the reusability quality of the prepared material to decrease costs. In the first cycle, Iron oxide prepared using Clove could remove 81.50% of Cd ^2+^ metal ions. The removal efficiency of tested material for Cd ^2+^ metal ions decreased to 72% after five cycles, as shown in Fig. 16c. This may be attributed to the decreased available reactive sites of iron oxide prepared using Clove^75^.

### Removal of Cd^2+^ metal ions from the industrial wastewater

An optimum dosage of iron oxide prepared using Clove was used to remove the Cd^2+^ metal ions in industrial wastewater in the Beni-Suef governorate, Egypt, in order to investigate its efficacy and economic feasibility in processing realistic wastewater containing Cd^2+^ metal ions in the presence of other competing ions such as, Cu^2+^, Zn^2+^ and Fe^2+^. Industrial effluent showed 5.50 mg/L of Cd^2+^ metal ions. Cd^2+^ metal ions could be removed with an efficiency of up to 98%. The results showed that iron oxide prepared using Clove could effectively remove Cd^2+^ metal ions from wastewater despite the presence of significant concentrations of competing ions (Cu^2+^, Zn^2+^, and Fe^2+^). The iron oxide prepared using Clove adsorbent had showed excellent potential for treating wastewater that contains realistic quantities of Cd^2+^ metal ions.

### Antibacterial assay

**Figure 17:**
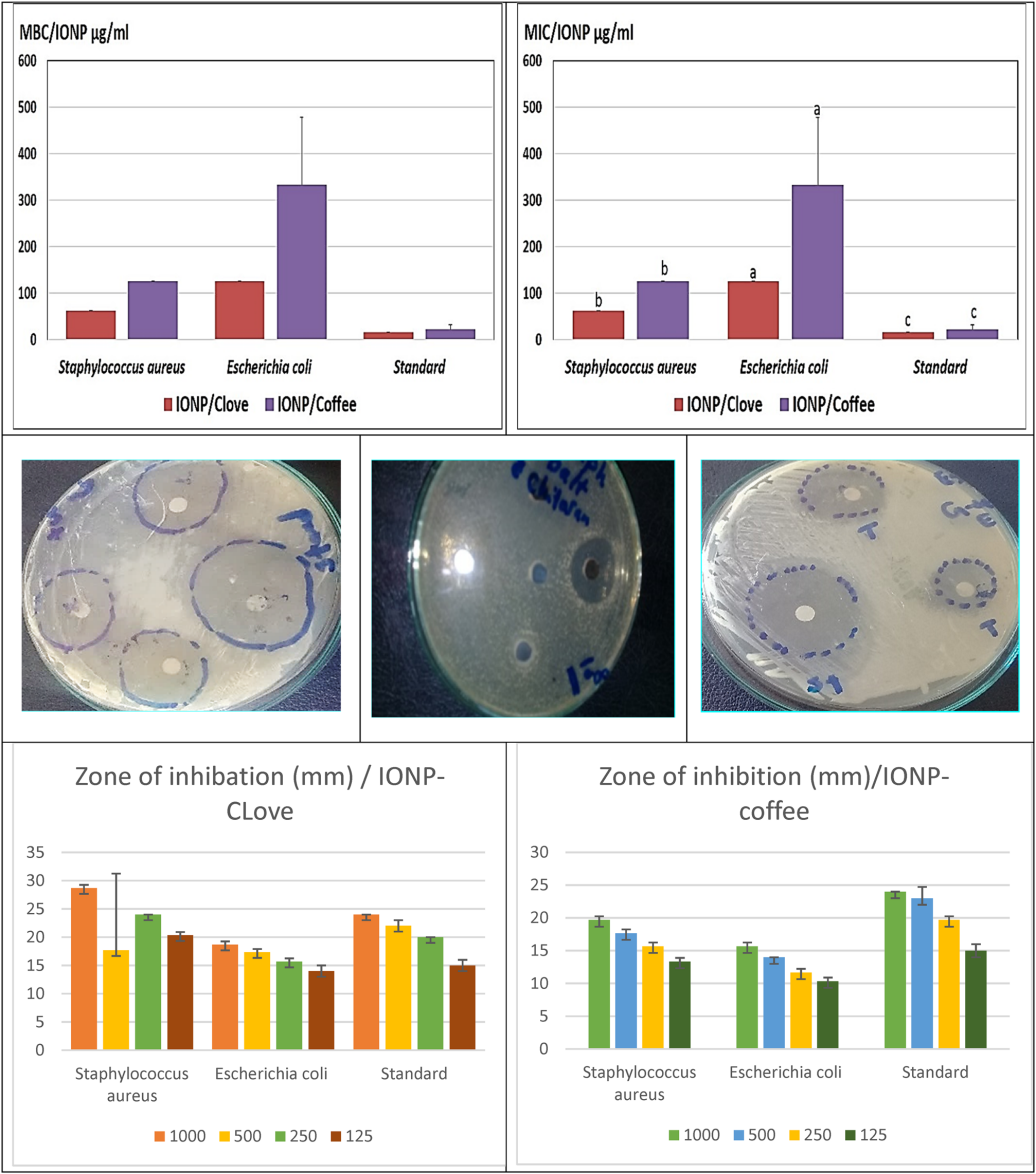
Antimicrobial activity of IONP of both Clove and g-Coffee extract against both Gram positive and Gram negative bacteria.

Figure 17 displays different plates of various strains of bacteria as Gram positive (*Staphylococcus aureus), and* Gram negative (*E-coli)* and shows the inhibition zone of each strain with variant concentrations of iron oxide nanoparticals (IONPs). The inhibition zones were measured in (mm) by Agar diffusion method. Overall, the measured diameters were different from one species to another. And as such, Fig. 17 is a bar chart that illustrates the calculated mean of the inhibition zone (mm) and the different concentrations of IONPs prepared using Clove or g-Coffee (1000, 500, 250, 125, 62.5 ug/ml) versus diverse species of bacteria.

In total, the effect of IONPs on the tested strains was uneven. First, regarding Gram positive, the *S. aureus* was the highest responded with higher activity for that’s of irone oxide nanoparticles prepared using Clove extract than g-Coffee one. Second, the response of *E coli* Gram negative was higher in also Clove extract one than g-Coffee extract. Furthermore, it is obtained that the inhibition zone was directly proportional to the concentration of IONPs in all investigated species. The highest inhibition zone was about 29 mm and was measured in *S. aureus*, but was 19.5 against *E. coli* for g-Clove estraxt, whereas approximately 20 and 16 mm was recorded for the same strains in the g-Coffee extract. It’s worth to mention that, the tested IONPs of both extracts was compared with Draxxin for Gram negative (32 mm) and Tylvalosin (29 mm) from Gram positive both were at the highest concentration, the results revealed that the effect of IONPs of both extracts was good against both Gram negative and positive species. From this prospective point, the investigated IONPs of both extracts could act as an efficient alternative for bacterial resistance than conventional antibiotics.

It should be noted that, the MIC and MBC of the investigated IONPs of both extracts versus the aforementioned bacterial species, as was shown in Fig. 17. Actually, the Fig. 17 reveals that the MIC value of the tested IONPs of both extracts was significantly distinct from one species to another, whereas the recorded values of MBC and MIC are similar as in *A. aureus* and *E-coli* for both Clove and g-Coffee which indicates the good efficacy of Clove extract. Figure 17 exhibits the maximum value of MIC was 62.5 µg/ml against *Staphylococcus aureus* and 125 µg/ml *against E. coli*. Where as were 125 and 250 µg/ml for a g-Coffee extract for the same organism, respectively. On the other hand, the value of MBC was recorded to be the same for the MIC data wich indicates the bactericidal activity of the tested extracts. The iron oxide antimicrobial activity was stated before by Iconaru et al.^111^ that’s stated the good antimicrobial activity of iron oxide.

The antibacterial IONP activity of both Clove and g-Coffee extacts; ROS production is one of the key mechanisms of IONP toxicity on bacterial cell activity^112^. In turn, ROS causes damage to the bacterial DNA molecules through a process known as genotoxicity^112^. A decrease in the activity of antioxidant system enzymes can result in an increase in ROS levels (SOD, catalase, and glutathione reductase)^113^ Amino (–NH), and carboxyl (–COOH) groups of proteins inside the bacterial cell, including those in enzymes, can bind to metal ions, causing deactivation or partial inhibition for the bacterial activity^114^. The integrity of the bacterial cell wall is also harmed by IONPs, as demonstrated in reference^112^ or with direct inhibition for the bacterial activity throgh the direct interaction bacterial cell membrane^114^. The integrity of the bacterial cell wall is also harmed by IONPs, as demonstrated in reference^112^. Antibiotic resistant bacteria observed in operating rooms may express less antibiotic resistance genes (ARGs) when exposed to IONPs.^115^ The activity of F0/F1-ATPase, the rate of H^+^ passage through the membrane, and the redox potential can all be affected by IONPs^115^. For nanoparticles with tiny diameters, a capability to prevent DNA replication by inactivating topoisomerase is described^116^. Additionally, IONPs have the ability to enter the cytoplasm and concentrate there, where they damage cell walls and create vacuoles^117^.

The mechanism of antibacterial action of Clove extract might be throgh affecting the bacterial cell integrity, harm cell membranes, and release nucleic acids and proteins that can impact bacterial development, prevent bacterial reproduction, and have antibacterial and sterilizing effects^118^ While caffeine, caffeic acid, and phenolic compounds were identified as the chemicals responsible for the antibacterial activity in earlier investigations^119^. Additionally, aromatic and phenolic substances have an antibacterial effect by changing the structure and function of the plasma membrane, as well as by impairing proton motive force and active transport^120^.

**Figure 18:**
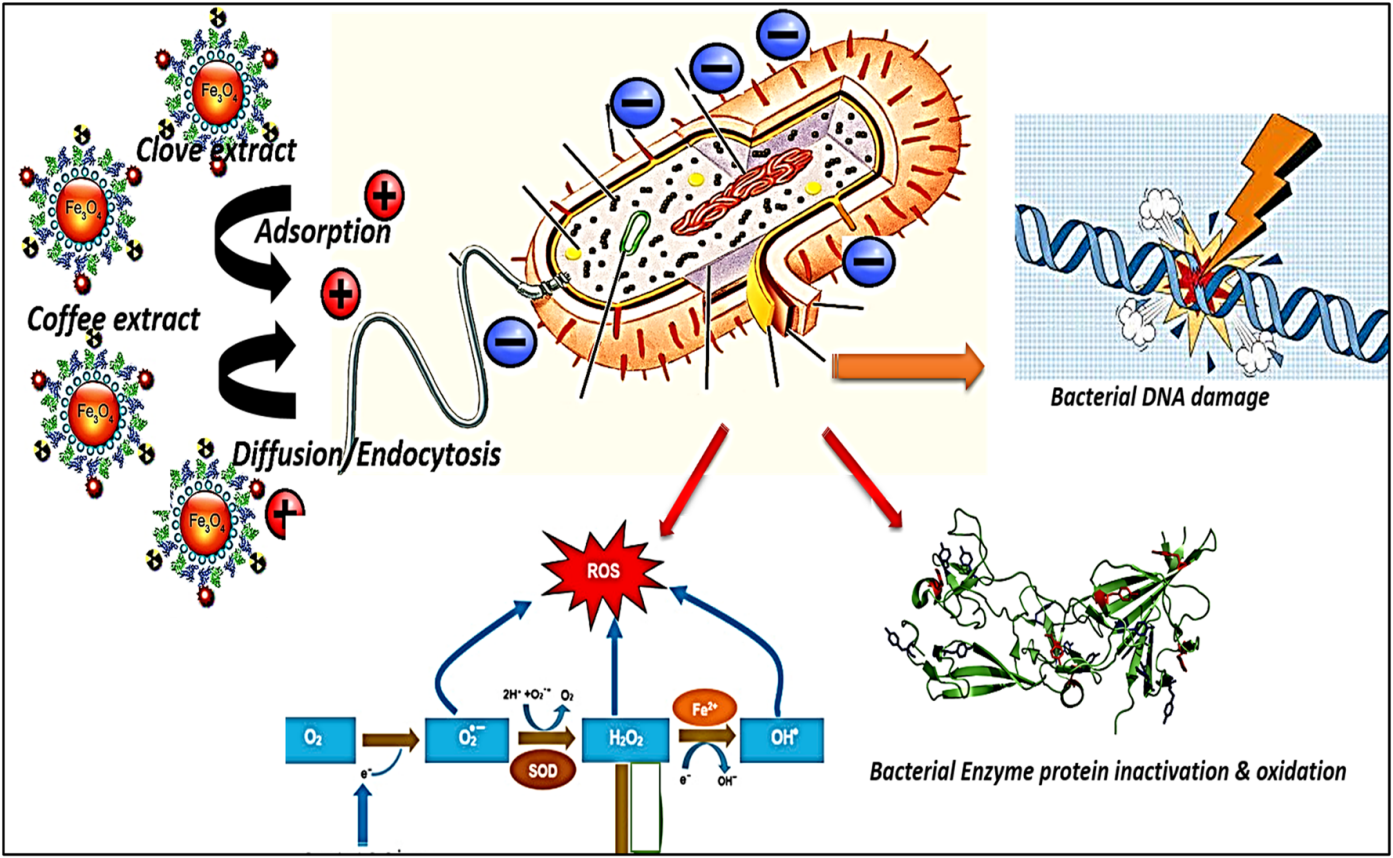
Schematic depicting the mechanisms of IONPs antibacterial activity of iron oxide nanoparticles prepared using Clove and g-Coffee extracs.

In conclusion, the studied IONPs prepared using g-Coffee or Clove extract may have an impact on various bacterial strains in a dose-dependent way and according to a specific pattern, with higher activity against Gram positive than Gram negative bacteria. The antibacterial activity of IONPs prepared using g-Coffee or Clove extract may have been caused by the spontaneous release of free radicals like ROS, in agreement with prior research. The many pores in the thin layer of peptidoglycan in Gram negative bacteria may have allowed IONPs to enter the cell, causing membrane disruption, the release of cell contents, and ultimately cell death. This is similar to how IONPs entered Gram positive bacteria as showed in Fig. 18.

## Conclusions

Iron oxide nanoparticles were synthesized using Clove and green g-Coffee extracts. Annealing at 550 °C improved the crystalline phase of the iron oxide nanoparticles. Adsorption isotherm models indicated that Sips isotherm, and Fritz-Schlunder isotherm are the best models to describe the adsorption of Ni^2+^ ions on iron oxide nanoparticles prepared by Clove and g-Coffee, respectively. While, Baudu isotherm, and Freundlich and Fritz-Schlunder isotherms are the best model to describe the adsorption of Cd^2+^ on iron oxide nanoparticles prepared using Clove and g-Coffee extract, respectively. The pseudo-second order was the best kinetic model (R^2^ = 0.99) to describe the adsorption of Cd^2+^ and Ni^2+^ ions on the iron oxide nanoparticles. It was found that a removal of 100% of Cd^2+^ and Ni^2+^ could be accomplished through adsorption with 0.05 g/L of iron oxide nanoparticles, an initial concentration of metal at 20 ppm, pH of 6, and a room temperature of 25 °C. The green iron nanoparticles obtained in this study have a comparatively high adsorption capacity, making them a promising contender for industrial applications. The antibacterial studies showed that iron nanoparticles prepared using Clove had effective bactericidal activity against the studied pathogens more than those prepared using g-Coffee. Where iron oxide prepared using Clove had a 62.5 μg/ml MIC against *S. aureus* and 125 against *E.coli*. Iron oxide prepared using g-Coffee were 125 and 150 μg/ml against *S. aureus* and *E. coli*. Iron oxide prepared using Clove inhibited *S. aureus* at 29 mm, *E.coli* at 19.5 mm, and those prepered using g-Coffee at 20 and 16 mm.

In conclusion, the findings of our research work may be considered as serious, talented, and novel, suggesting that iron oxide preparing via green synthesis might be engaged as cost-effective and an efficient adsorbent for heavy metal ions adsorption. Iron oxide has wonderful potential for wastewater treatment. Further research must be undertaken on iron oxide for various polutants removal, as evaluated in this investigation, to transfer from the laboratory scale to the manufacturing.

## Supplementary Information

Supplementary Information.

## Data Availability

The datasets used and/or analysed during the current study available from the corresponding author on reasonable request.
