# Supplementary material for: Green synthesis and characterization of iron oxide nanoparticles for the removal of heavy metals (Cd2+ and Ni2+) from aqueous solutions with Antimicrobial Investigation
Source: Sci Rep. 2023 May 4;13:7227. doi: 10.1038/s41598-023-31704-7 (PMC10160056; doi:10.1038/s41598-023-31704-7)
Supplement: Supplementary file 1 — Supplementary Information. [file 41598_2023_31704_MOESM1_ESM.docx]

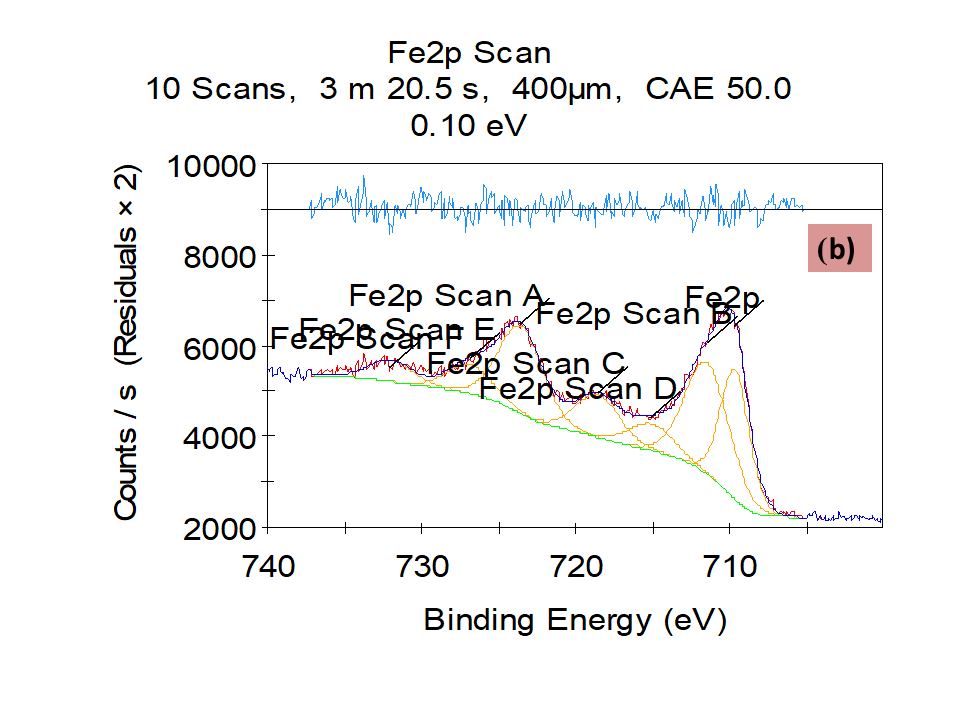

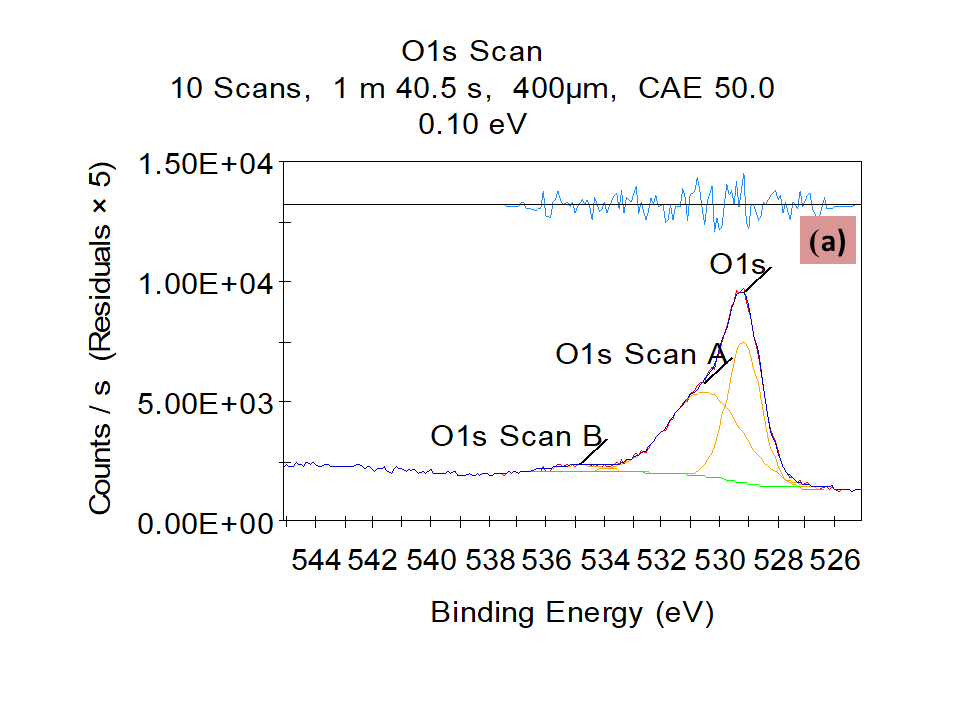

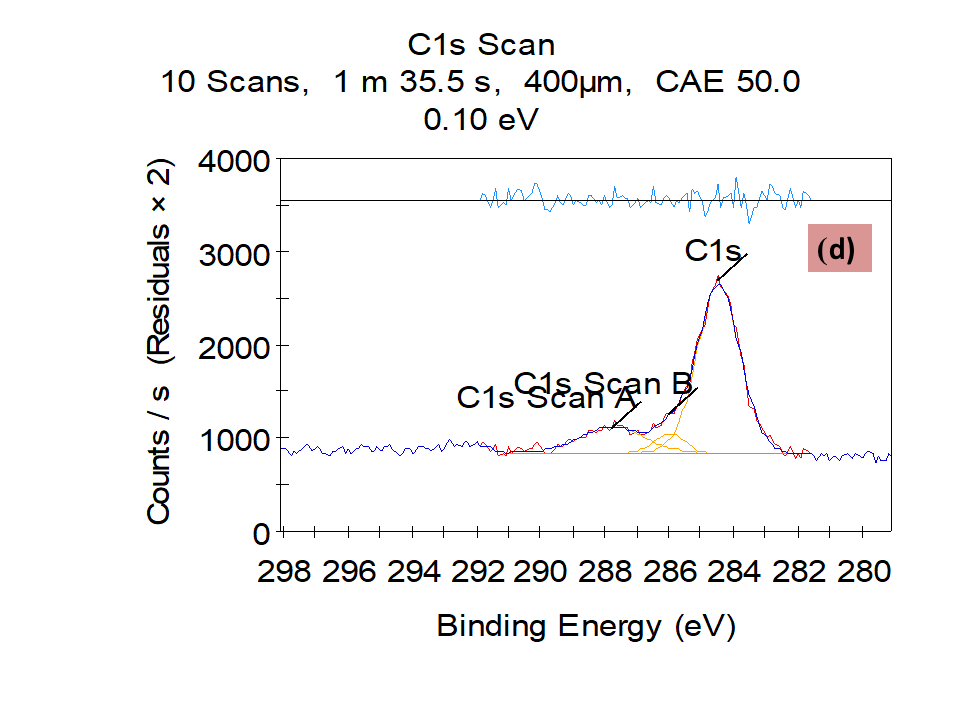

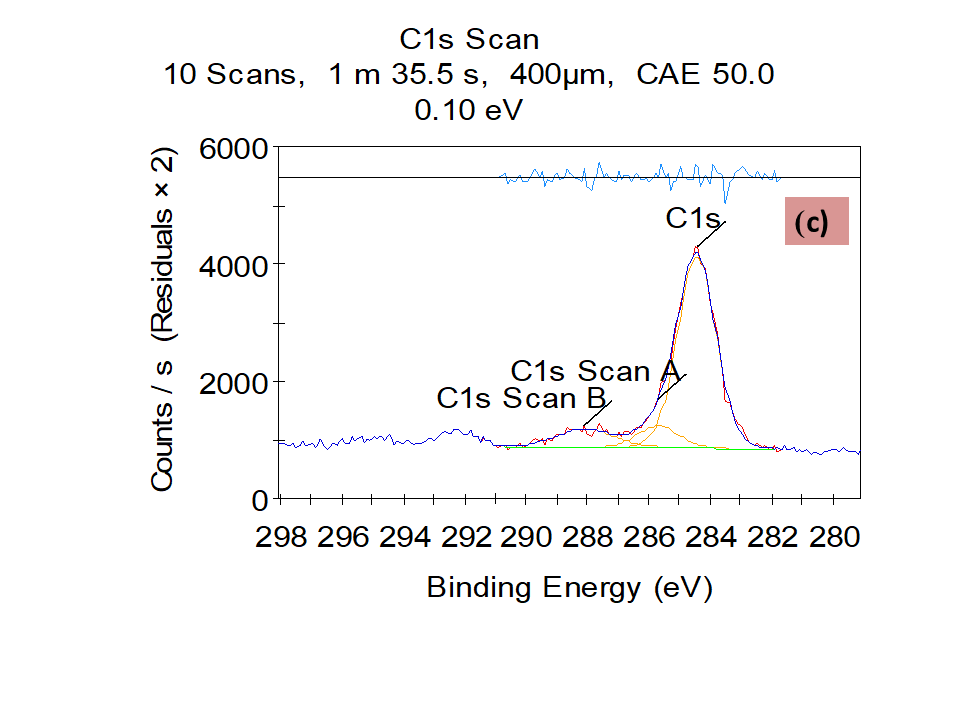

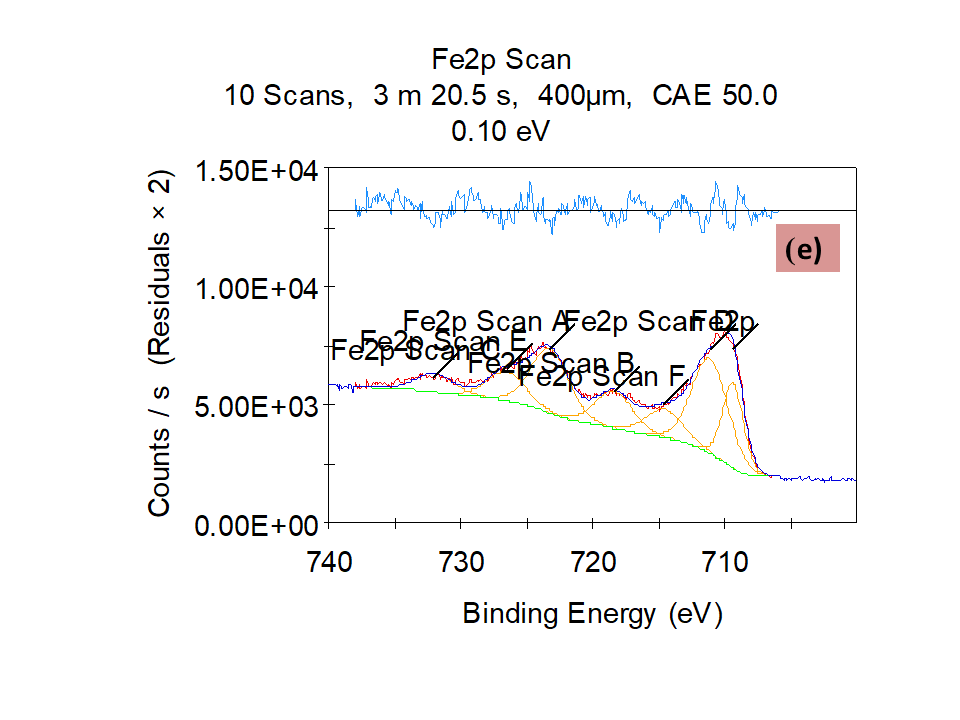


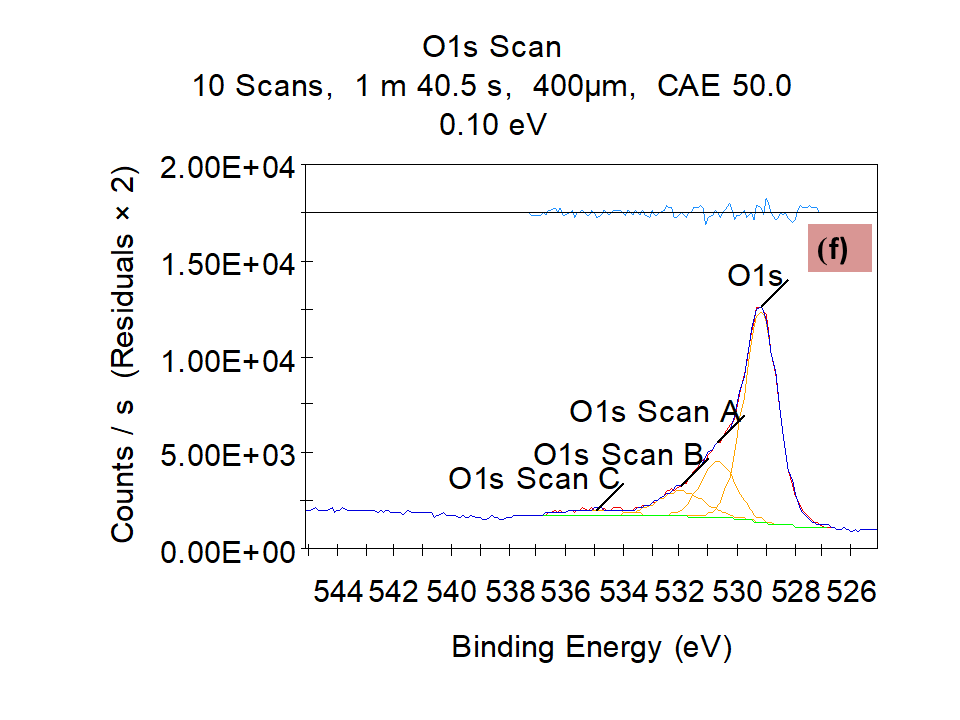
**Figure S1.** XPS spectra of the iron oxide nanoparticles (a-c) using Clove extracts and (d-f) using g-Coffee extract.

| a | 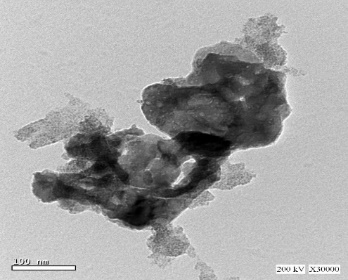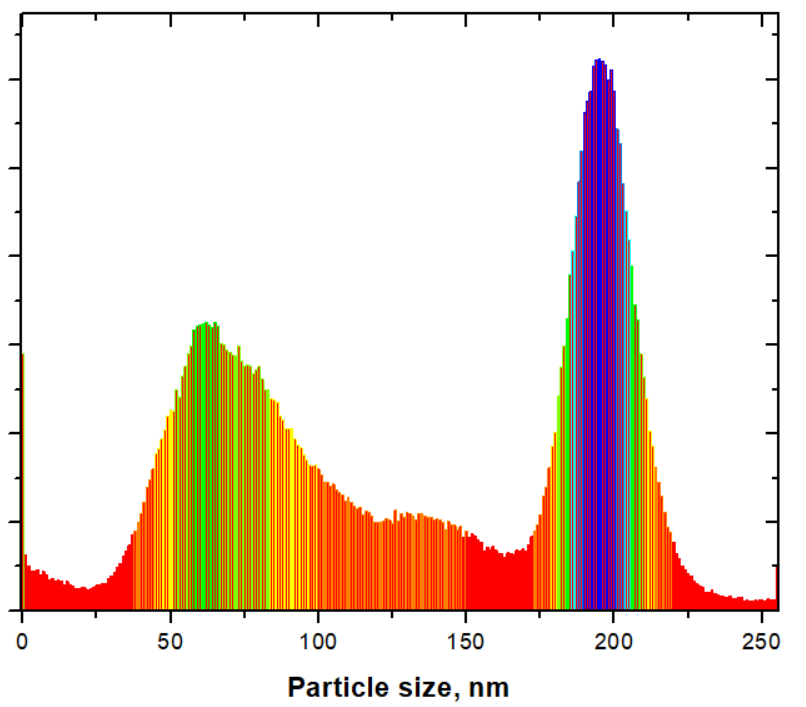 | 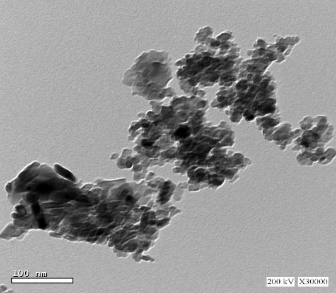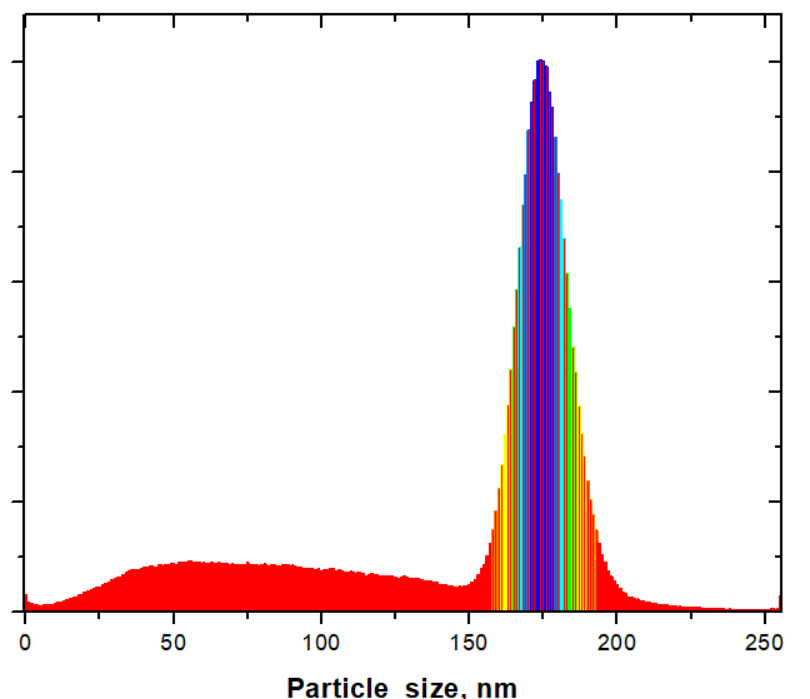 |
| --- | --- | --- |
| b | 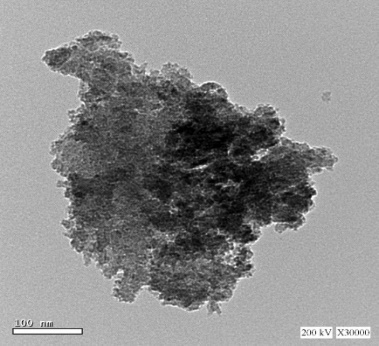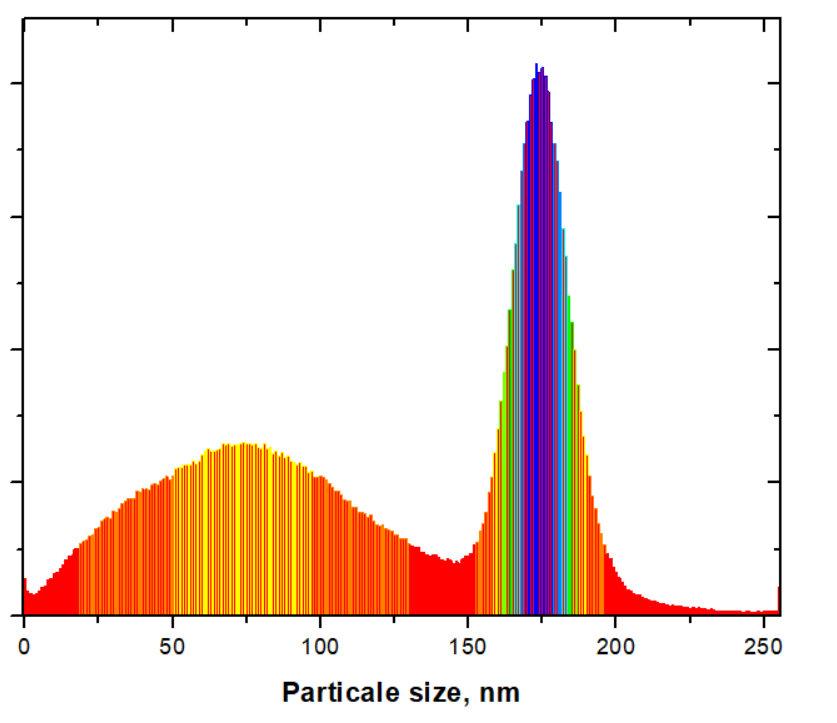 | 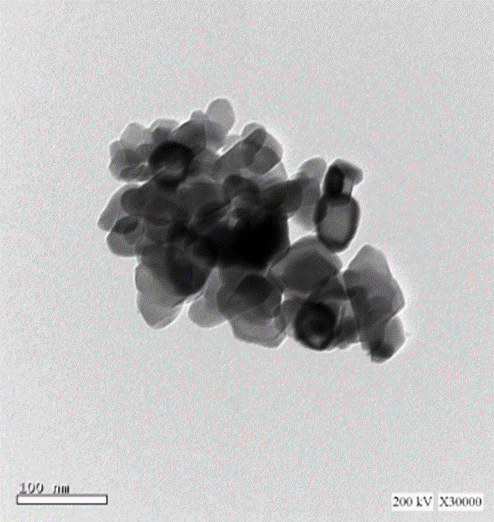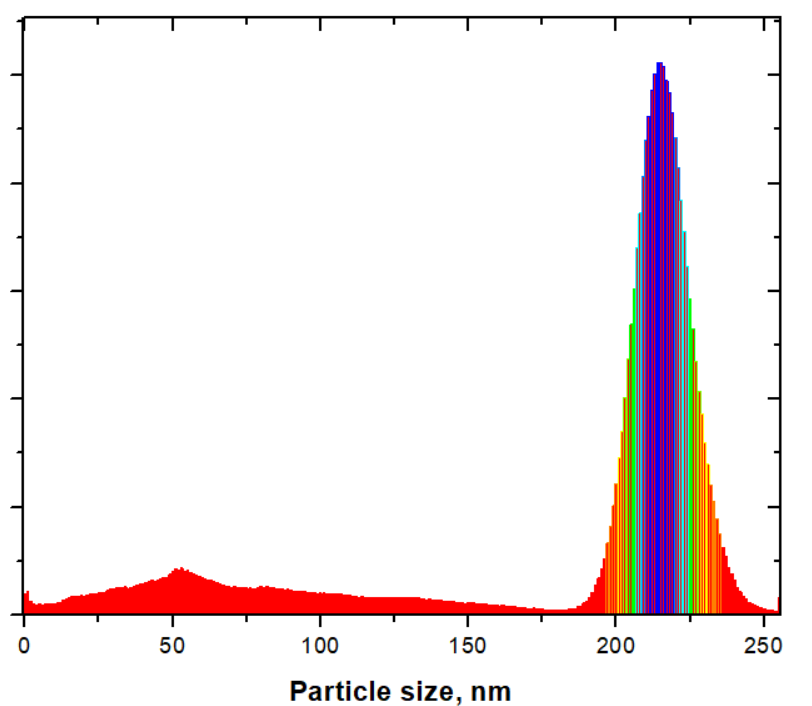 |

**Figure S2.** The particle size distribution of prepared iron oxide nanoparticles using a) clove, and b) coffee extract at room temperature and annealed at 550 °C.

|  |  |
| --- | --- |

**Figure S3.** The effect of sorbent dosage on the adsorption of Cd (II) and Ni (II).

|  |  |
| --- | --- |

**Figure S4.** The effect of Cd (II) and Ni (II) concentration on the adsorption.

|  |  |
| --- | --- |

**Figure S5.** The effect of time on the adsorption of Cd (II) and Ni (II).
